# Measurements of Na^+^-occluded intermediates during the catalytic cycle of the Na^+^/K^+^-ATPase provide novel insights into the mechanism of Na^+^ transport

**DOI:** 10.1016/j.jbc.2022.102811

**Published:** 2022-12-17

**Authors:** Santiago E. Faraj, Wanda M. Valsecchi, Mariela Ferreira-Gomes, Mercedes Centeno, Elina Malén Saint Martin, Natalya U. Fedosova, Juan Pablo FC. Rossi, Mónica R. Montes, Rolando C. Rossi

**Affiliations:** 1 Universidad de Buenos Aires, Facultad de Farmacia y Bioquímica, Departamento de Química Biológica, Buenos Aires, Argentina; 2 Consejo Nacional de Investigaciones Científicas y Técnicas (CONICET) – Universidad de Buenos Aires, Instituto de Química y Fisicoquímica Biológicas "Prof. Alejandro C. Paladini" (IQUIFIB), Buenos Aires, Argentina; 3 Department of Biomedicine, Aarhus University, Aarhus, Denmark

**Keywords:** Albers–Post model, cation transport intermediates, epigallocatechin-3-gallate, enzyme kinetics, enzyme mechanism, membrane transport, Na^+^/K^+^-ATPase, sodium occlusion, sodium transport, EGCg, epigallocatechin-3-gallate, QWC, quenching-and-washing chamber

## Abstract

The Na^+^/K^+^-ATPase is an integral plasma membrane glycoprotein of all animal cells that couples the exchange of intracellular Na^+^ for extracellular K^+^ to the hydrolysis of ATP. The asymmetric distribution of Na^+^ and K^+^ is essential for cellular life and constitutes the physical basis of a series of fundamental biological phenomena. The pumping mechanism is explained by the Albers–Post model. It involves the presence of gates alternatively exposing Na^+^/K^+^-ATPase transport sites to the intracellular and extracellular sides and includes occluded states in which both gates are simultaneously closed. Unlike for K^+^, information is lacking about Na^+^-occluded intermediates, as occluded Na^+^ was only detected in states incapable of performing a catalytic cycle, including two Na^+^-containing crystallographic structures. The current knowledge is that intracellular Na^+^ must bind to the transport sites and become occluded upon phosphorylation by ATP to be transported to the extracellular medium. Here, taking advantage of epigallocatechin-3-gallate to instantaneously stabilize native Na^+^-occluded intermediates, we isolated species with tightly bound Na^+^ in an enzyme able to perform a catalytic cycle, consistent with a genuine occluded state. We found that Na^+^ becomes spontaneously occluded in the *E*1 dephosphorylated form of the Na^+^/K^+^-ATPase, exhibiting positive interactions between binding sites. In fact, the addition of ATP does not produce an increase in Na^+^ occlusion as it would have been expected; on the contrary, occluded Na^+^ transiently decreases, whereas ATP lasts. These results reveal new properties of *E*1 intermediates of the Albers–Post model for explaining the Na^+^ transport pathway.

The Na^+^/K^+^-ATPase (EC 7.2.2.13) is an integral glycoprotein of the plasma membrane of all animal cells that couples the exchange of intracellular Na^+^ for extracellular K^+^ to the hydrolysis of ATP (1, 2, 3). The asymmetric distribution of Na^+^ and K^+^ is essential for cellular life and constitutes the physical basis of a series of fundamental biological phenomena such as the conservation of cell volume, the generation and propagation of the nerve impulse, and the transport of glucose and amino acids.

According to the Albers–Post model (Fig. 1), the Na^+^/K^+^-ATPase undergoes phosphorylation and dephosphorylation reactions and alternates between two main conformations, *E*1 and *E*2, which expose the transport sites to the intracellular and extracellular sides of the membrane, respectively (4, 5, 6, 7, 8, 9). The pumping mechanism involves the presence of gates and must not allow exposing the transport sites simultaneously to both sides of the membrane; this necessarily implies the existence of ion-bound states in which both gates are closed at the same time, that is, occluded states.

**Figure 1:**
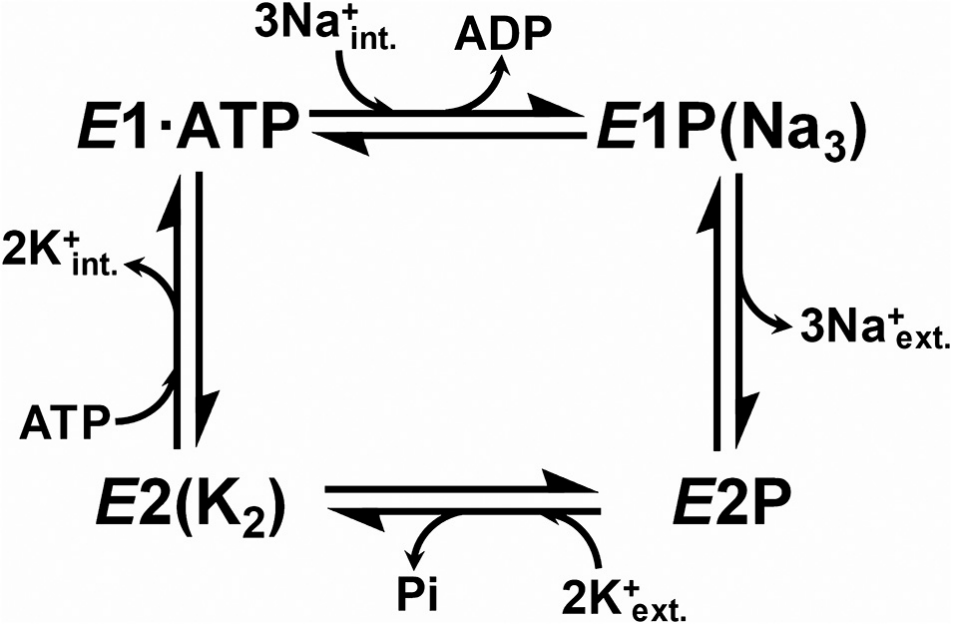
**Simplified scheme of the catalytic cycle of the Na**^**+**^**/K**^**+**^**-ATPase.** The binding of three intracellular Na^+^ ions to the *E*1·ATP state of the Na^+^/K^+^-ATPase triggers phosphorylation, and Na^+^ becomes trapped into the transmembrane domain in the *E*1P(Na_3_) intermediate. After the conformational transition, Na^+^ ions are released to the extracellular medium, with the formation of *E*2P. The binding of two extracellular K^+^ produces the rapid dephosphorylation of the enzyme, with the formation of the K^+^-occluded state *E*2(K_2_). The binding of ATP to *E*2(K_2_) promotes the release of K^+^ to the intracellular medium, leading to the formation of *E*1·ATP. Note the use of brackets to indicate cation occlusion.

The ease of measuring potassium-occluded intermediates and assessing their contribution to the normal Na^+^/K^+^-ATPase reaction cycle (10, 11, 12, 13, 14, 15, 16, 17) contrasts with the systematic failure to measure sodium occlusion in native preparations, given the much higher kinetic instability of Na^+^-occluded states. This prompted the search for optimal conditions based on the hypothesis that sodium is occluded in *E*1P (18, 19, 20). Thus, it was possible to measure Na^+^ occlusion in phosphorylated enzyme pretreated with *N*-ethylmaleimide or α-chymotrypsin (21) or using creatine–ATP (22). An analog of *E*1P.ADP formed in the presence of oligomycin, ADP, and AlF_4_ was also found to occlude sodium (23). These results reaffirmed the original hypothesis that sodium is occluded concomitantly with the formation of *E*1P. As an exception, occluded sodium was also measured in dephosphorylated enzyme in the presence of oligomycin (24). However, none of the aforementioned conditions allow assessing the contribution of Na^+^-occluded states during the normal transport cycle.

It is evident that sodium deocclusion needs to be effectively quenched for states with occluded Na^+^ to be captured and measured. In the case of cation occlusion, it is mandatory to preserve the structure of the pump, and denaturing agents cannot be used (25). Instead, other physicochemical procedures are required, such as a sudden drop in temperature (which is not sufficient for this specific purpose) or the use of a stabilizing agent at neutral pH. We took advantage of the finding that epigallocatechin-3-gallate (EGCg) stabilizes all the intermediates of the pump in the *E*1 conformation (26) in a way similar to that of oligomycin (27, 28). In contrast to oligomycin, EGCg has three orders of magnitude higher water solubility, which allows millimolar concentrations of the agent in the solution. Such high concentrations, combined with its relatively high affinity for the Na^+^/K^+^-ATPase, ensure prompt binding of EGCg to the enzyme and its applicability in quench-flow experiments.

Here, we report the direct measurement of Na^+^ occlusion in transport intermediates of the Na^+^/K^+^-ATPase. We determined that the Na^+^/K^+^-ATPase is capable of spontaneously occluding sodium in a cooperative manner. We also found that Na^+^-occluded intermediates represent a minor fraction of the total enzyme during ATP hydrolysis and that their level is restored upon ATP depletion.

## Results

### EGCg as a tool: conformational changes, equilibrium interactions, and quenching capacity

In the next few paragraphs, we characterize the ability of EGCg to stabilize the *E*1 conformations of the enzyme and, when added to the washing solution of a rapid filtration procedure, to be used as a quencher preventing the loss of Na from the occluded intermediates.

It has been reported that EGCg inhibits Na^+^/K^+^-ATPase activity by stabilizing the *E*1 conformation of the pump. In agreement with Ochiai *et al.* (26), we found that ATPase activity decreases with [EGCg] with a *K*_0.5_ of 1.10 ± 0.03 μM (Fig. S1). Using eosin to assess the *E*2 → *E*1 conformational change in the Na^+^/K^+^-ATPase, we found that when EGCg was added to Rb^+^-bound enzyme, it shifted the equilibrium to the *E*1 conformation (Fig. S2) with an observed kinetic coefficient of about 0.132 ± 0.003 s^−1^, regardless of the concentration of EGCg in the tested range.

**Figure 2:**
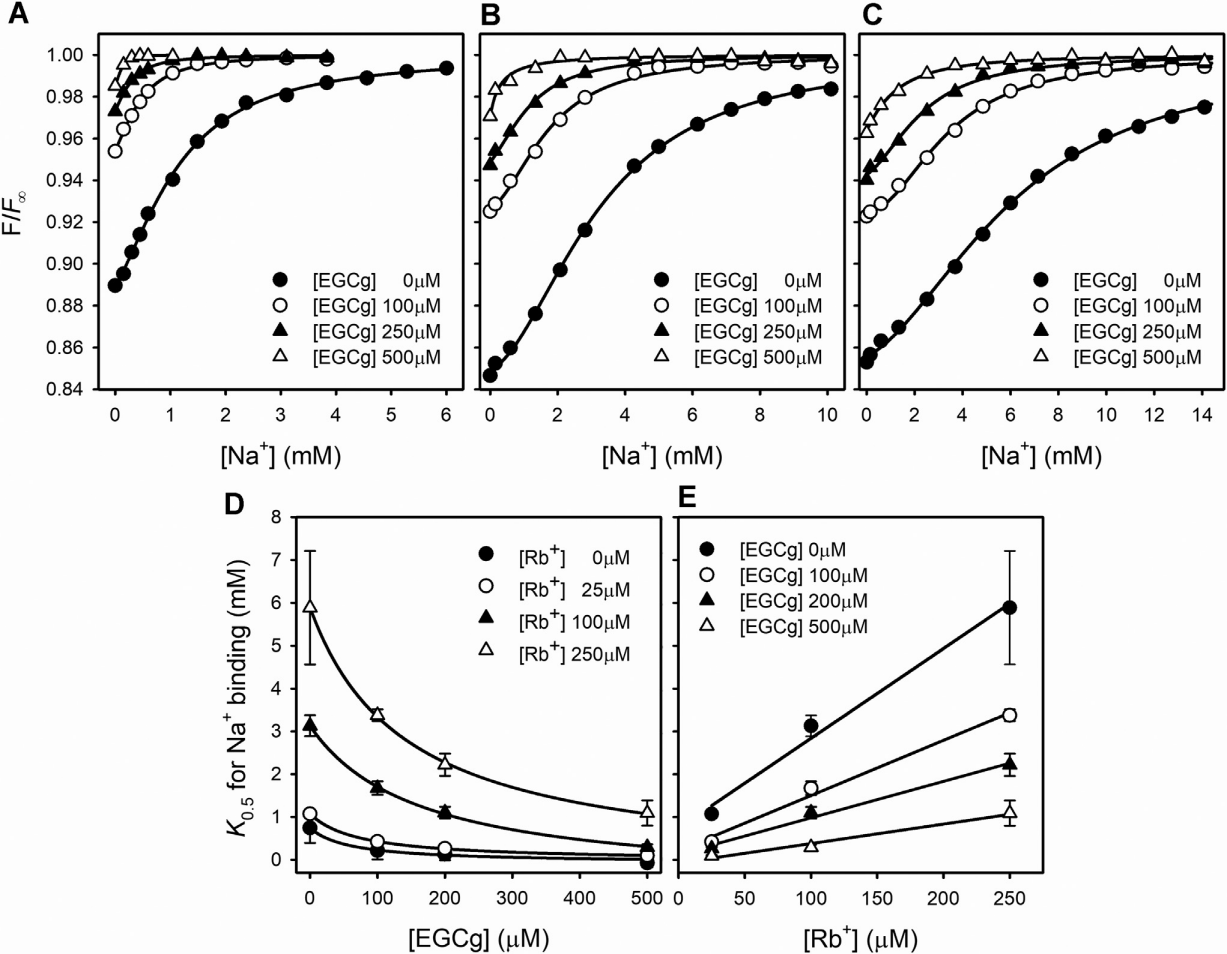
**Effect of EGCg on Na**^**+**^**affinity of Na**^**+**^**/K**^**+**^**-ATPase.** Eosin fluorescence in an enzyme suspension at varying [NaCl], in the presence of (*A*) 25, (*B*) 100, and (*C*) 250 μM RbCl. All reaction media also contained 25 mM imidazole/HCl (pH 7.4 at 25 °C) and 0.25 mM EDTA. Fluorescence is shown as *F*/*F*_*∞*_ to correct for EGCg quenching. Na^+^-binding *K*_0.5_ values obtained from fitting a rational function (Equation S3) to the data in *A*–*C* as a function of (*D*) [EGCg] or (*E*) [RbCl]. Error bars represent ±1 SE. EGCg, epigallocatechin-3-gallate.

To assess the effect of EGCg on the enzyme affinity for Na^+^, we measured eosin fluorescence under equilibrium conditions, at varying concentrations of EGCg and Rb^+^ (Fig. 2).

In Figure 2, *A*–*C*, we show normalized results (F*/F*_*∞*_) of the original data (Fig. S3) as described in the Experimental procedures section. Normalized eosin fluorescence increases with [EGCg] in the absence of Na^+^ and further increases with [Na^+^] along sigmoid curves (29) indicating a shift toward the *E*1 conformation that involves the binding of more than one Na^+^ ion.

There is a shift to the left of the curves as [EGCg] increases, for all Rb^+^ concentrations tested, with a corresponding decrease in the values of *K*_0.5_ for Na^+^ (Fig. 2*D*). As shown by Esmann (30), these values increase with [Rb^+^] (Fig. 2*E*). We can conclude that EGCg stabilizes states of the Na^+^/K^+^-ATPase in the *E*1 conformation and increases the affinity of the enzyme for Na^+^.

**Figure 3:**
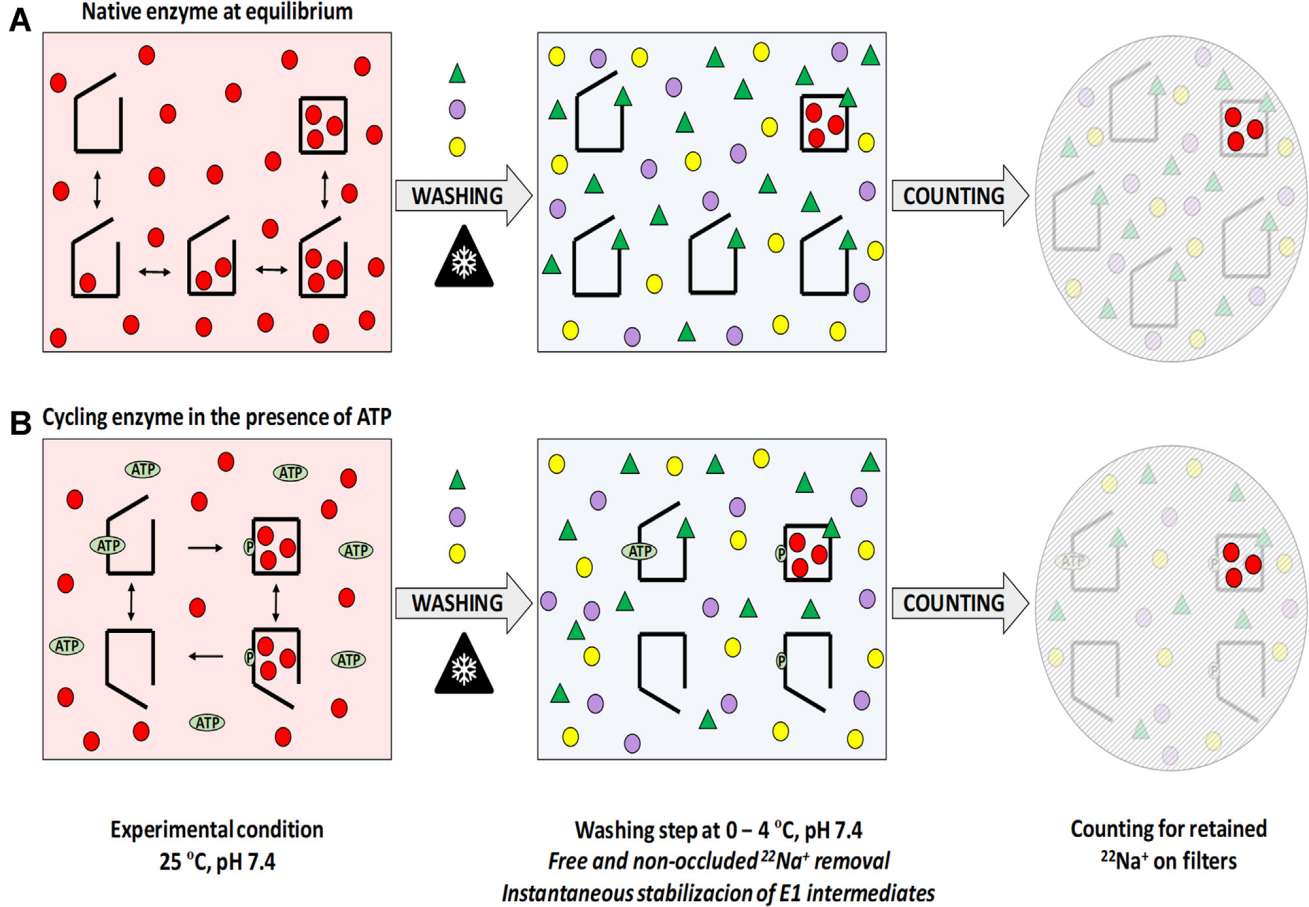
**Measuring occluded sodium.** The Na^+^/K^+^-ATPase was incubated with ^22^Na^+^ () in the absence (*A*) or presence (*B*) of ATP. In *A*, species with none, one, two, or three sodium ions remain at equilibrium with Na^+^-occluded forms. In *B*, the enzyme cycles through the Na^+^-ATPase activity forming Na^+^-occluded and phosphorylated intermediates until all ATP is depleted. In order to determine the amount of occluded Na^+^ at a certain time during the process, the reaction was injected into a quenching-and-washing chamber with a cellulose-ester filter, which quantitatively retains the enzyme, and washed with an ice-cold buffer containing 1 mM EGCg () in order to instantaneously quench the *E*1 species present in the reaction. The washing solution also contains high concentrations of unlabeled Na^+^ () and K^+^ () to prevent the formation of new ^22^Na^+^-occluded species. After washing, filters were dried and ^22^Na^+^ was counted.

We investigated if EGCg works as a stabilizing agent of Na^+^/K^+^-ATPase Na^+^-occluded species. Our procedure uses a rapid filtration technique in which EGCg is typically present only in the washing solution (see Experimental procedures section). The underlying hypothesis is that the binding of EGCg to the enzyme instantaneously stabilizes those intermediates containing occluded Na^+^ (Fig. 3). The washing solution also contains unlabeled Na^+^ and K^+^ in concentrations high enough to prevent the formation of new ^22^Na^+^-occluded species (Fig. S4).

**Figure 4:**
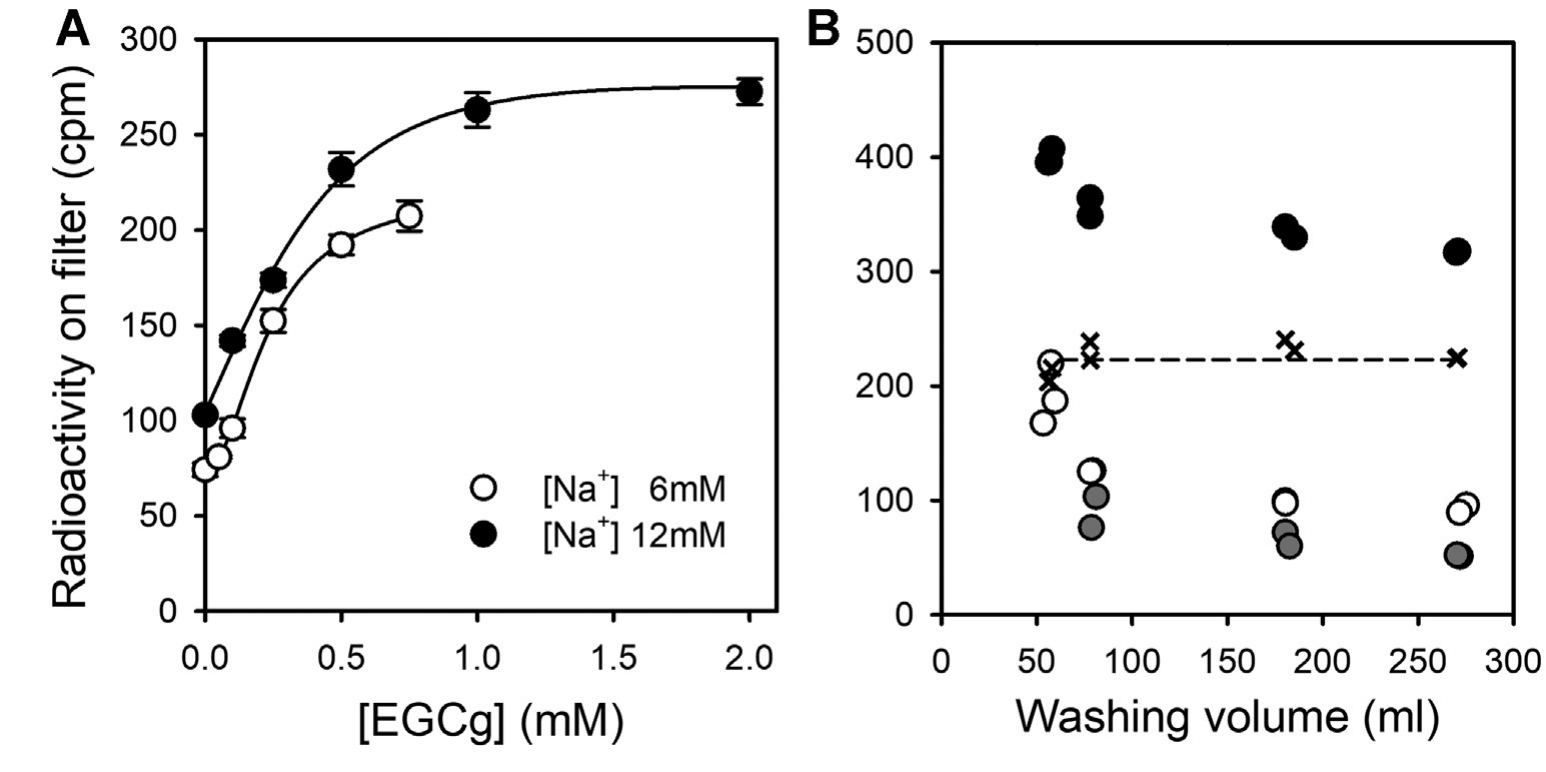
**Effect of EGCg on Na**^**+**^**retention.***A,* Na^+^/K^+^-ATPase was incubated at 25 °C with 6 or 12 mM ^22^Na^+^ for a time long enough as to reach equilibrium. Enzyme suspensions were injected into the quenching-and-washing chamber and washed with different concentrations of EGCg. Error bars represent ±1 SD. *Continuous lines* were included to guide the eye. *B,* remaining radioactivity as a function of washing solution volume. The enzyme in equilibrium with 1 mM ^22^Na^+^ was injected into the quenching-and-washing chamber and washed with varying volumes of solution with (,) or without () 1 mM EGCg. The difference between radioactivity values obtained for the active enzyme with and without EGCg is shown (×). A control experiment with inactivated enzyme was also included (). All reaction media also contained 25 mM imidazole/HCl (pH 7.4 at 25 °C) and 0.25 mM EDTA. EGCg, epigallocatechin-3-gallate.

To test the optimal concentration of EGCg in the washing solution, the amount of ^22^Na^+^ retained on the filters was measured as a function of [EGCg] for reaction media with 6 mM or 12 mM NaCl (Fig. 4*A*). The values increased with the concentration of EGCg tending to a plateau. From these results, we decided to supplement washing solutions with 1 mM EGCg in order to quench reactions.

To determine the volume of solution necessary to wash out free ^22^Na^+^, we performed an experiment where the filters were washed with different volumes of ice-cold buffer immediately after the reaction mixture was injected into the quenching-and-washing chamber (QWC). Results in Figure 4*B* show that using around 100 ml of washing solution (2.5 s at a flow rate of 40 ml s^−1^) is enough to wash out most of the free radioactivity on the filters; note that 2.3 × 10^6^ cpm were injected into the QWC in each determination. These results led us to establish a volume of 250 ml to ensure proper washing. The control without EGCg in the washing solution produced much lower values, being the difference between the values obtained with and without EGCg in the washing solution constant with the washing volume (Fig. 4*B*). This difference must mainly be caused by the presence of Na^+^-occluded species, and its invariance denotes that these species are stable during the washing step. These results show that combining the cooling-and-washing procedure with the use of EGCg as a stabilizing agent allows the detection of Na^+^-occluded intermediates. It is noteworthy that the use of thermally inactivated enzyme produces lower ^22^Na^+^ retention values than intact enzyme when incubated with a very high concentration of unlabeled Na^+^ (Fig. S5)—accounting only for a fraction of the unspecific retention of radioactivity—and would lead to an overestimation of the amount of occluded Na^+^ if it were used as blanks.

We also verified that retained Na^+^ is proportional to the amount of Na^+^/K^+^-ATPase and that at least part of it is unspecific, as evinced by an experiment carried at 10 mM K^+^ (Fig. S6).

### The Na^+^/K^+^-ATPase occludes Na^+^ in the absence of ATP

**Figure 5:**
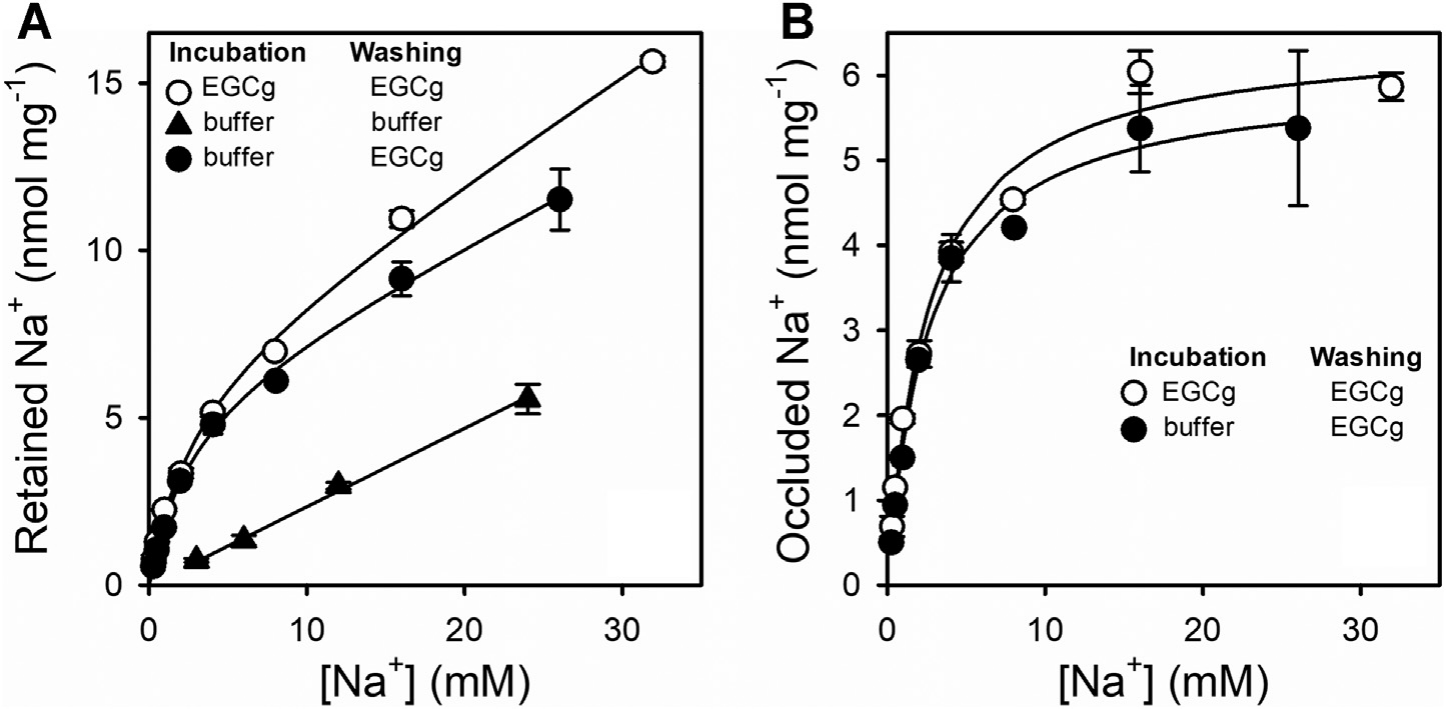
**Equilibrium values of occluded Na**^**+**^**.** Na^+^/K^+^-ATPase was incubated with or without 1 mM EGCg, at varying concentrations of Na^+^. Once equilibrium was reached, enzyme suspensions were injected into the quenching-and-washing chamber and washed with a solution with or without 1 mM EGCg. All reaction media also contained 25 mM imidazole/HCl (pH 7.4 at 25 °C) and 0.25 mM EDTA. *A,* retained Na^+^. *Continuous lines* are fittings of Equation 1 to the data. *B,* values of occluded ^22^Na^+^ were obtained by subtracting unspecific retention from data in *A*. *Continues lines* are the fitting of simple rectangular hyperbolas to the data. Error bars represent ±1 SD. EGCg, epigallocatechin-3-gallate.

To determine the affinity for Na^+^ of the Na^+^/K^+^-ATPase to form Na^+^-occluded states (*cf*., Fig. 3*A*), we measured occluded Na^+^ as a function of [Na^+^] (Fig. 5). In addition, the enzyme was incubated in the presence of EGCg to investigate whether this inhibitor modifies the enzyme affinity for Na^+^. We observed that the Na^+^/K^+^-ATPase spontaneously occludes Na^+^ and that its affinity for the cation is not affected by EGCg. A control experiment omitting EGCg in the washing solution was also included, showing a linear increase of retained Na^+^, which suggests its unspecific nature, insufficient however to be used as a blank (Fig. S5).

Data could be described by

(1) $$Retained\;Na^{+}=\frac{Occ_{\max}\left[Na^{+}\right]}{K_{0.5}+\left[Na^{+}\right]}+m\left[Na^{+}\right]$$

where *Occ*_max_ is the maximal Na^+^ occlusion capacity of the enzyme and *m* is the slope of unspecific Na^+^ binding. For active and EGCg-inhibited enzymes, respectively, we found *Occ*_max_ values of 6.0 ± 0.2 and 6.5 ± 0.2 mM and *K*_0.5_ for Na^+^ of 2.6 ± 0.3 and 2.6 ± 0.2 mM (Fig. 5*B*). These results show that, provided that EGCg is included in the washing step, similar values are obtained when the enzyme is incubated with or without EGCg. This fact indicates that our procedure allows quantitative detection of almost all Na^+^-occluded species and that EGCg does not change the affinity for Na^+^ or promote any further occlusion beyond that that spontaneously occurs. Considering the number of nucleotide sites in the preparations used in this work (see Experimental procedures section), the maximum occlusion values are consistent with a stoichiometry of three Na^+^ ions per enzyme unit. The hyperbolic shape of the specific component seems to contrast with the proposal of cooperativity between Na^+^-binding sites.

**Figure 6:**
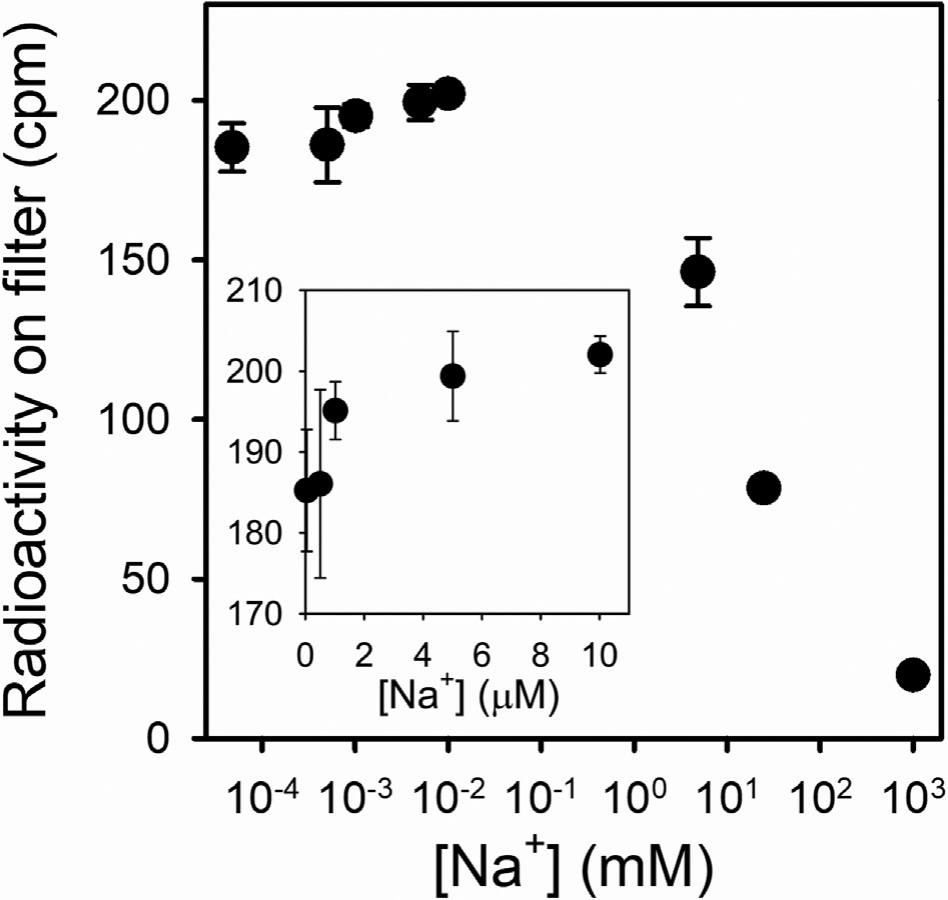
**Cooperativity in**^**22**^**Na**^**+**^**occlusion.** Na^+^/K^+^-ATPase in equilibrium with 2 × 10^6^ cpm ml^−^^1^ of Na^+^ and varying concentrations of total NaCl (beginning at 48 nM) were injected into the quenching-and-washing chamber and washed with a solution containing 1 mM EGCg. All reaction media also contained 25 mM imidazole/HCl (pH 7.4 at 25 °C) and 0.25 mM EDTA. The *inset* shows a zoom at low [Na^+^]. Error bars represent ± 1SD. EGCg, epigallocatechin-3-gallate.

However, an experiment performed at very low concentrations of Na^+^ revealed the existence of significant interactions between the Na^+^ sites (Fig. 6). The enzyme was incubated with carrier-free ^22^Na^+^ (about 48 nM) plus increasing concentrations of unlabeled NaCl. It is evident that, because of the decrease of the specific activity of ^22^Na^+^, the radioactivity on the filters decreases as the concentration of unlabeled Na^+^ increases indefinitely. However, the addition of up to 10 μM unlabeled Na^+^ leads to an increase in radioactivity on the filters (Fig. 6, *inset*), which showed to be significant (*p* < 0.01). Such an increase should not happen if the Na^+^-binding sites were identical and independent, and it reveals the existence of positive interactions between these sites that more than compensate for the drop in specific activity of ^22^Na^+^. Therefore, the hyperbolic shape of the curves in Figure 5*B* is only apparent, and sigmoidicity should be observed at a very low [Na^+^].

### A model to explain observations in equilibrium experiments

**Figure 7:**
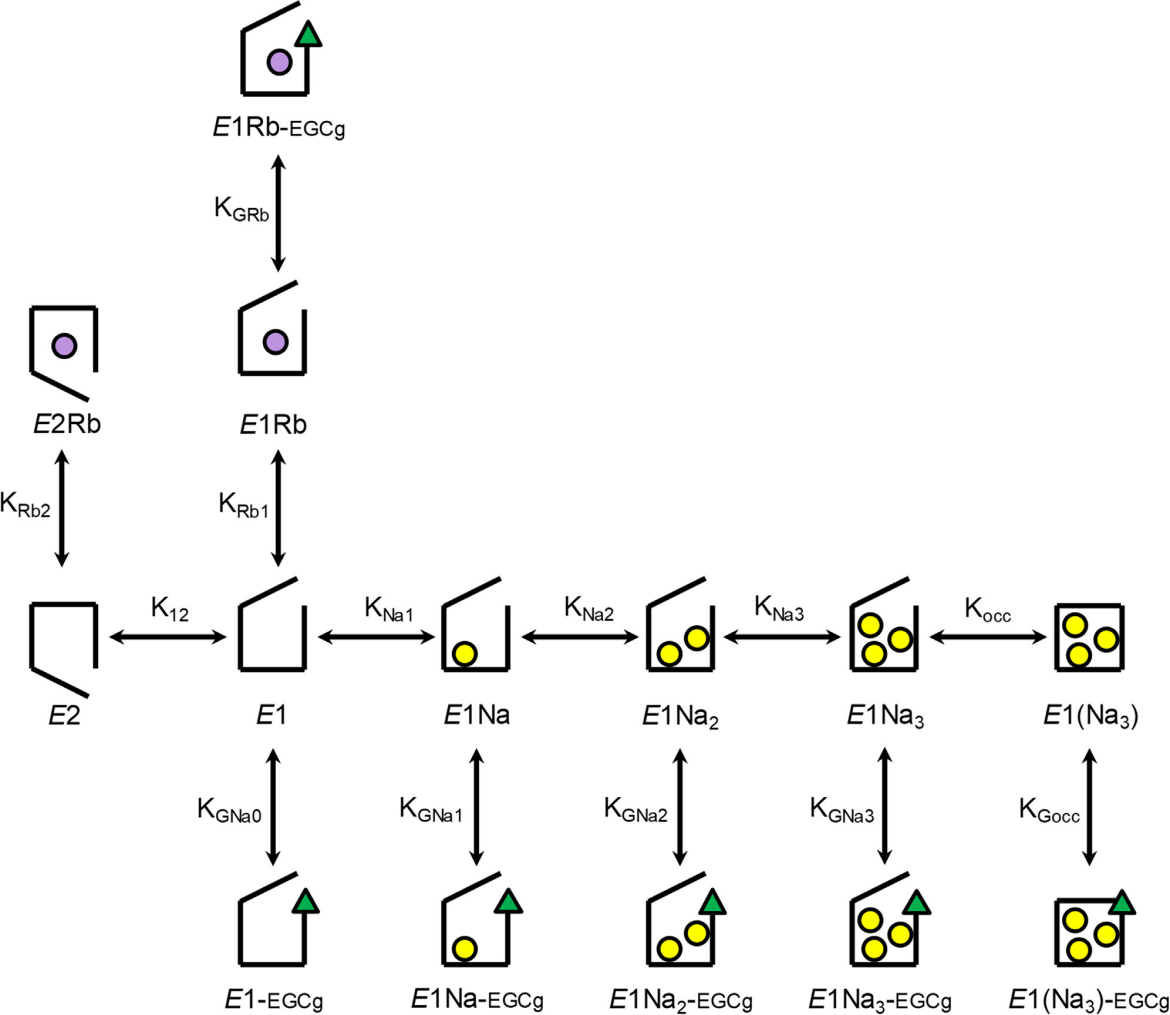
**Equilibrium Na**^**+**^**occlusion model.** Scheme of the equilibrium model of the Na^+^/K^+^-ATPase at equilibrium in the presence of EGCg (), Na^+^ (), and K^+^ (). All species in the *E*1 conformation are considered to equally bind eosin, and consequently, equally contribute to the observed fluorescence. Occluded Na^+^ is shown between *brackets*. EGCg, epigallocatechin-3-gallate.

The results of eosin fluorescence (Fig. 2) and Na^+^ occlusion experiments (Fig. 5) are contradictory: EGCg increased the affinity for Na^+^ in the eosin experiments with *K*_0.5_ values in the submillimolar range, whereas, in occlusion experiments, the affinity for Na^+^ appeared to be an order of magnitude lower and not significantly affected by EGCg. This apparent discrepancy is easily resolved if we hypothesize that, while the formation of the occluded state increases with the number of sites occupied by Na^+^, the binding of a single Na^+^ is enough to promote the *E*1 conformation. The model in Figure 7, which does not intend a detailed quantitative description of the experimental observations, is based on this idea.

**Figure 8:**
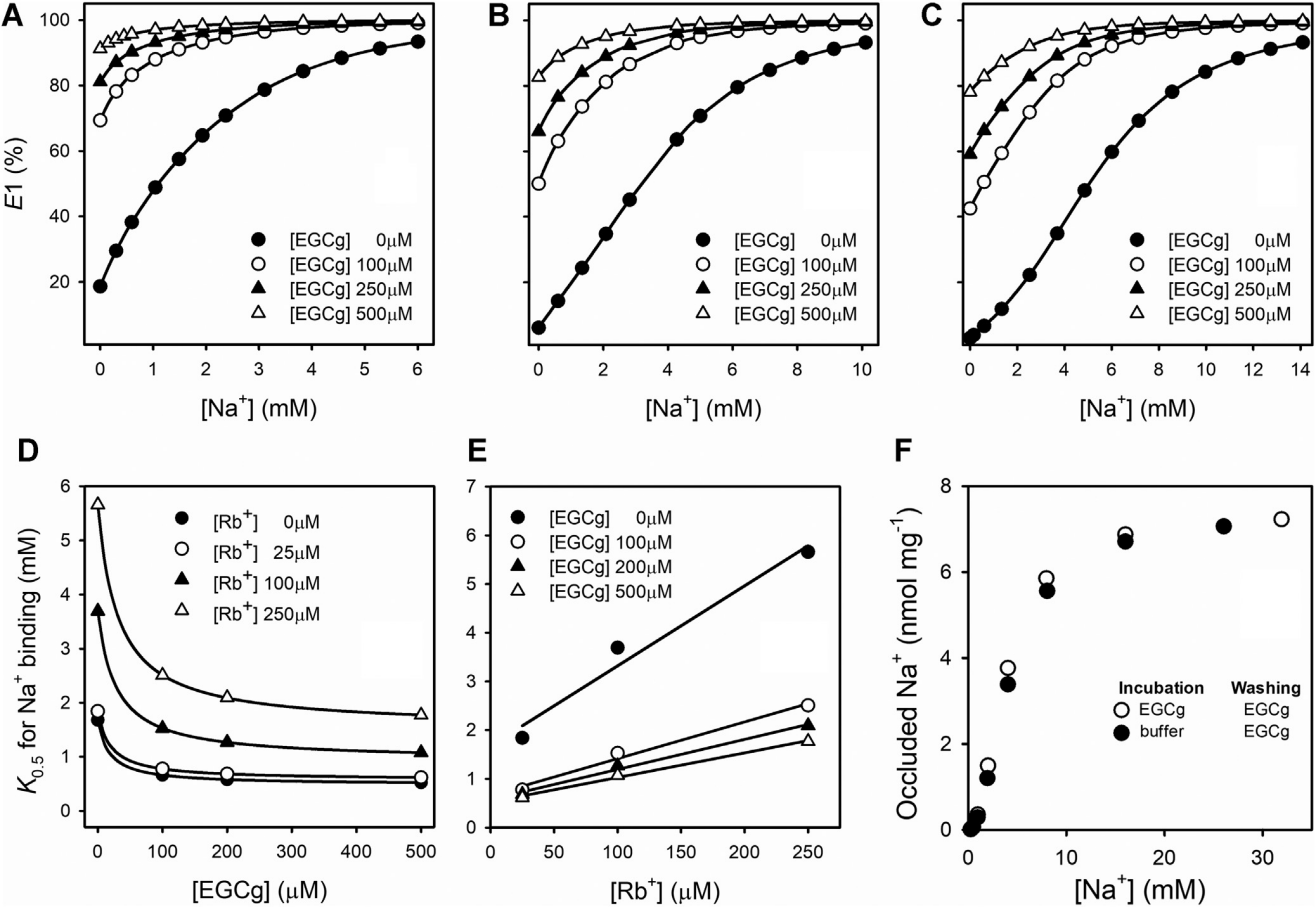
**Equilibrium Na**^**+**^**binding and occlusion model.** The model shown in Figure 7 was simulated using the following parameters values: *K*_*12*_ = 0.25, *K*_*Na1*_ = 0.36 mM, *K*_*Na2*_ = 20 mM, *K*_*Na3*_ = 75 mM, *K*_*occ*_ = 100, *K*_*G0*_ = 0.015 mM, *K*_*GNa1*_ = 0.017 mM, *K*_*GNa2*_ = 0.023 mM, *K*_*GNa3*_ = *K*_*Gocc*_ = 0.014 mM, *K*_*Rb1*_ = 0.86 mM, *K*_*GRb*_ = 0.0012 mM, and *K*_*Rb2*_ = 0.0015 mM. Eosin fluorescence in an enzyme suspension at varying [NaCl], in the presence of (*A*) 25, (*B*) 100, and (*C*) 250 μM RbCl. Na^+^-binding *K*_0.5_ values obtained from fitting a rational function (Equation S3) to the data in *A*, *B*, and *C* as a function of (*D*) [EGCg] or (*E*) [RbCl]. *F,* values of occluded ^22^Na^+^ at varying [NaCl].

The model assumes that the Na^+^/K^+^-ATPase exists in two main conformations, *E*1 and *E*2, and that the sequential binding of three Na^+^ to *E*1 leads to a state, *E*1(Na_3_), that is the only one capable of becoming occluded. This does not exclude the possibility of the existence of occluded species with less than three Na^+^, but these would represent only small fractions of the occluded Na^+^. The K^+^-congener Rb^+^ can also bind to the enzyme, in either of the two conformations. For the sake of simplicity of the model, only one Rb^+^ ion was considered ignoring the known stoichiometry of two Rb^+^ per enzyme unit. EGCg can bind to all states in the *E*1 conformation, regardless of whether its sites are empty or occupied by Na^+^ or Rb^+^. In general (Fig. 8), the model adequately explains (i) the effect of Na^+^, which increases the fluorescence of eosin through curves that are the more sigmoid, the higher the concentration of Rb^+^ (*cf*., Fig. 2, *A*–*C*); it also allows to explain the interaction between Na^+^ sites observed in Figure 6; (ii) the effect of Rb^+^, which decreases both the fluorescence of eosin and the affinity of the enzyme for Na^+^ (*cf*., Fig. 2); and (iii) the effect of EGCg, which increases the affinity for Na^+^ in eosin fluorescence experiments (for any [Rb^+^]) but keeps it unchanged in Na^+^ occlusion experiments (*cf*., Fig. 5); it also explains why EGCg promotes the *E*1 conformation even in the absence of Na^+^ (*cf*., Fig. 2, *A*–*C*).

### Effects of Rb^+^ and Mg^2+^ on occluded Na^+^

**Figure 9:**
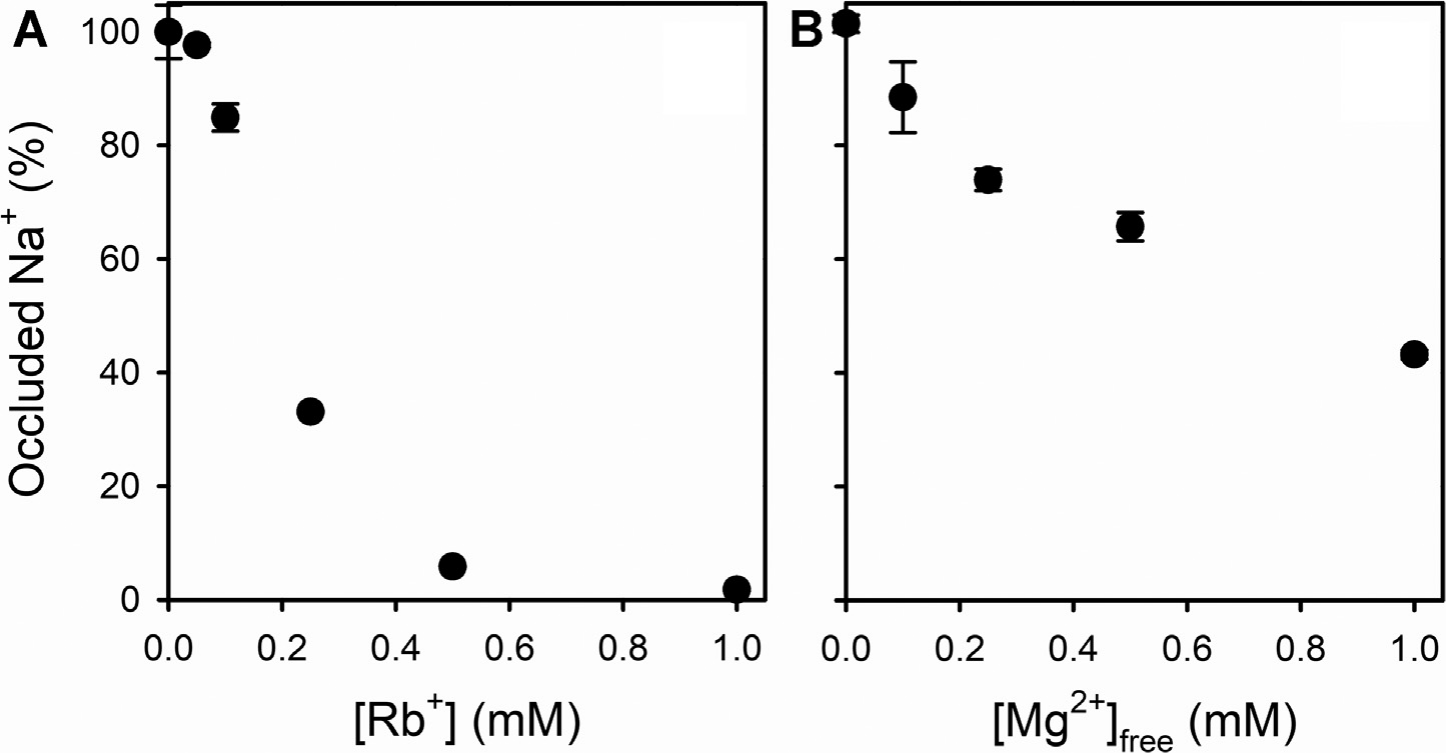
**Effect of Rb**^**+**^**and Mg**^**2+**^**on occluded Na**^**+**^**.** Equilibrium Na^+^ occlusion (as percentage of the total change in cpm) at 6 mM NaCl as a function of (*A*) Rb^+^ or (*B*) free Mg^2+^ concentrations. All reaction media also contained 25 mM imidazole/HCl (pH 7.4 at 25 °C) and 0.25 mM EDTA. Error bars represent ±1 SD.

As a K^+^ congener, Rb^+^ can bind to the cation transport sites, which should lead to a decrease in the affinity of the Na^+^/K^+^-ATPase for Na^+^ (*cf*., Fig. 2*E*). A natural consequence of this competitive effect is that increasing Rb^+^ concentrations decrease the amount of occluded Na^+^ (Figs. 9*A* and S7*A*). The model in Figure 7 also predicts this effect (not shown). The sigmoid shape of the curve reflects the binding of more than one Rb^+^ per enzyme unit.

Like Rb^+^, Mg^2+^ also interferes with Na^+^ binding (31, 32, 33) and causes a decrease in occluded Na^+^ (Figs. 9*B* and S7*B*). Crystallographic studies show that Mg^2+^ can also bind to the cation transport sites (34), even though it is not transported by the Na^+^/K^+^-ATPase.

### Na^+^ occlusion during the Na^+^-ATPase cycle of the Na^+^/K^+^-ATPase

**Figure 10:**
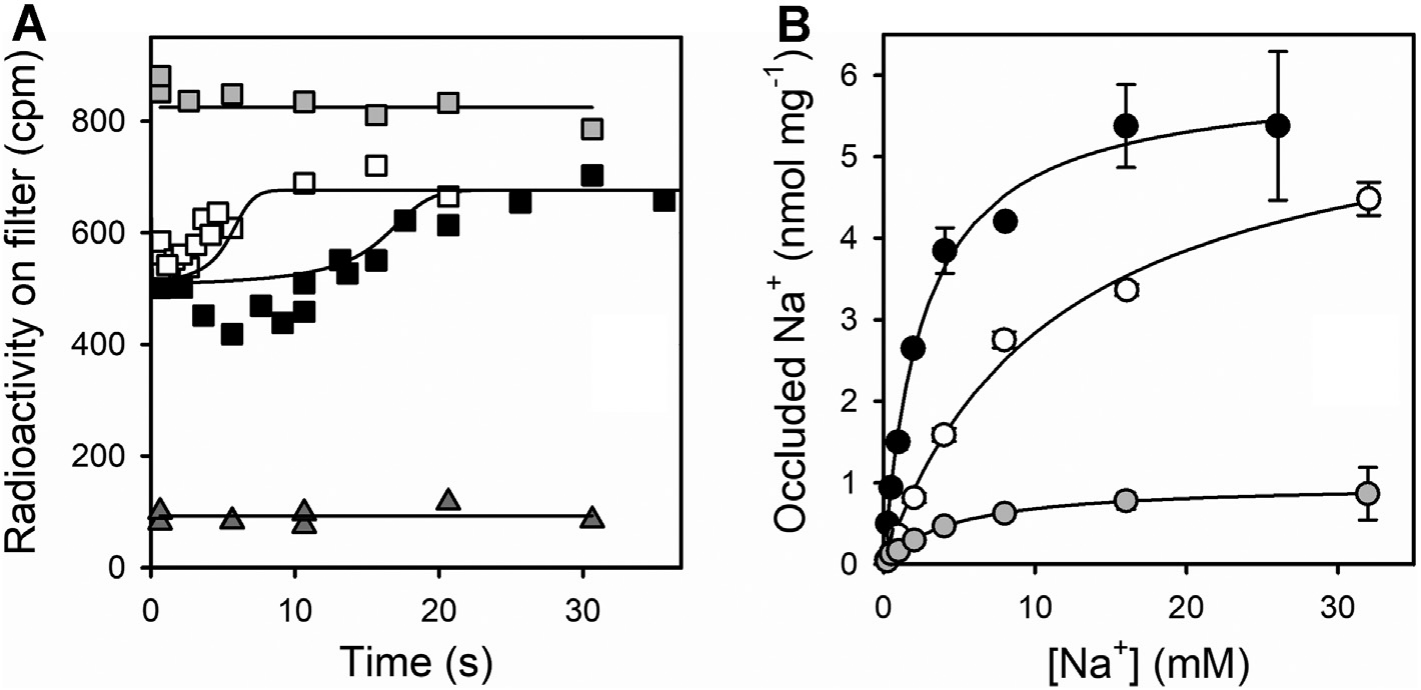
**Na**^**+**^**occlusion during ATP hydrolysis.***A,* time courses of Na^+^ occlusion upon addition of a mixture of MgCl_2_ and ATP to an enzyme suspension equilibrated with ^22^Na^+^. Final concentrations after addition of ATP + Mg^2+^ were 200 μg of protein per ml, 6 mM ^22^Na^+^, 0.5 mM free Mg^2+^, and 5 μM () or 15 μM () ATP. Control experiments with active () and inactivated enzyme () in the absence of ATP + Mg^2+^ were also included. *Continuous lines* are the best fitting of the model shown in Figure 11 for the parameters shown in Table 1. *B,* comparison between Na^+^ occlusion in steady-state and equilibrium experiments. Occluded Na^+^ 2 s after adding a mixture of MgCl_2_ and ATP to an enzyme suspension equilibrated with ^22^Na^+^ (note that 2 s are enough to reach steady state, as shown in Fig. S9*C*). Final concentrations were 200 μg of protein per ml, 0.5 mM free Mg^2+^, and 25 μM ATP (). Equilibrium spontaneous occlusion when the enzyme was incubated with () or without () 0.5 mM free Mg^2+^ is also shown (these latter results are from Fig. 5*B*). *Continuous lines* represent the best fitting of a rectangular hyperbola to the data. Error bars represent ±1 SD. All reaction media also contained 25 mM imidazole/HCl (pH 7.4 at 25 °C) and 0.25 mM EDTA.

Up to this point, we have shown that nonphosphorylated states of Na^+^/K^+^-ATPase can occlude Na^+^. It is a well-known fact that the enzyme can perform ATPase activity in the absence of K^+^, the Na^+^-ATPase activity cycle, at a rate that is only about 5% of its maximal activity in the presence of K^+^. To evaluate sodium occlusion as a transport step in the catalytic cycle, we measured its time course during the Na^+^-ATPase activity of the pump (*cf*., Fig. 3*B*). Results in Figure 10*A* show that, after the addition of ATP and Mg^2+^, occluded sodium transiently decreases and then increases as ATP is depleted.

**Figure 11:**
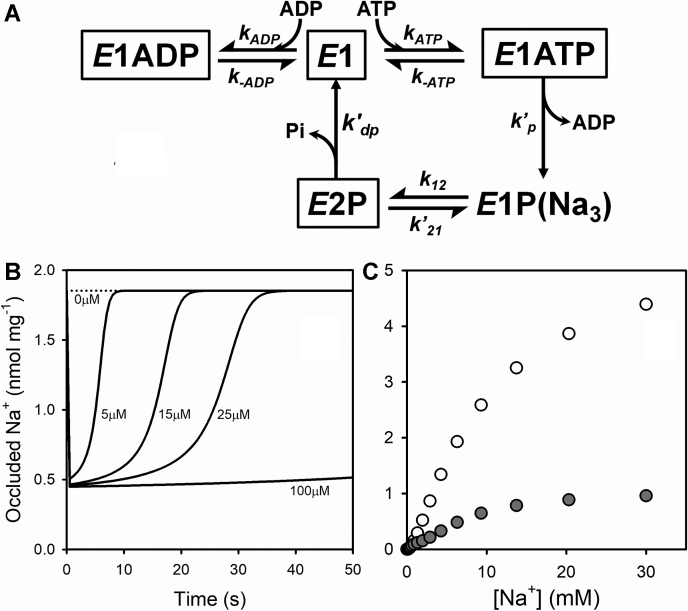
**Model of the Na**^**+**^**-ATPase cycle.***A,* this scheme is a simplified model in which each species shown within a *box* represents the sum of several species in rapid equilibrium with each other. *B,* simulations of the time course of occluded Na^+^ for 0.5 μM enzyme in the presence of 6 mM NaCl and varying concentrations of ATP (shown next to each curve). *C,* simulation of occluded Na^+^ 2 s after the beginning of the reaction with 0.5 μM enzyme and 25 μM ATP () and equilibrium spontaneous occlusion when the simulation was performed with no ATP (). Parameter values used for simulations are shown in Table 1.

It is noteworthy that the addition of ATP, which activates the Na^+^-ATPase cycling mechanism, allowing the formation of *E*1P(Na_3_), does not produce an increase in Na^+^ occlusion. On the contrary, occluded sodium transiently decreases as long as ATP is present. The explanation for this transient decrease could be that during the Na^+^-ATPase reaction cycle (see the model in Fig. 11*A*), the enzyme redistributes between a larger number of conformations (mainly in the *E*2P state), which do not hold occluded sodium. Consequently, the addition of ATP should lead to a decrease in occluded sodium, a condition that lasts until the complete depletion of ATP and the establishment of a new equilibrium. In this experiment, we did not attempt to monitor the pre–steady-state phase (the phosphorylation reaction) given its high speed and the interval analyzed.

As shown in Figure 10*A*, the higher the [ATP], the longer it takes for the same amount of enzyme to consume it and to reach the new equilibrium level of occluded Na^+^, which is significantly lower than the initial one because of the presence of Mg^2+^ (see Fig. 9*B* for the effect of Mg^2+^).

We measured steady-state occluded Na^+^ 2 s after the addition of ATP to the enzyme suspension pre-equilibrated with different concentrations of ^22^Na^+^ (Fig. S9*C*). The results are compared (Fig. 10*B*) with those of equilibrium experiments performed in media free of ATP and Mg^2+^ or containing 0.5 mM free Mg^2+^. The value of *K*_0.5_ for Na^+^ in the presence of 0.5 mM Mg^2+^ decreased from 11.1 ± 1.9 mM under equilibrium conditions to 4.5 ± 3.2 mM in steady state. Because of the presence of Mg^2+^, both the steady-state and equilibrium experiments show lower affinities for Na^+^ than in the absence of Mg^2+^ (*K*_0.5_ for Na^+^ of 2.6 ± 0.3, *closed circles* in Figs. 5*B* and 10*B*). Note that the maximum value of occluded Na^+^ in the absence and presence of Mg^2+^ remains unchanged (6.0 ± 0.2 *versus* 6.0 ± 0.4 nmol mg^−1^). More interestingly, steady-state occluded sodium reaches a maximum value much lower than the equilibrium curves (1.0 ± 0.2 nmol mg^−1^) (Fig. 11).

The scheme in Figure 11*A* is a simplified version of the Albers–Post model of the Na^+^-ATPase cycle, including the new findings reported in this article (see Fig. S8 for a more detailed description). States within boxes stand for the sum of every possible Na^+^-bound species, considered in rapid equilibrium with one another. Note that besides *E*1P(Na_3_), which exclusively exists as a state with occluded sodium, some species within the *E*1, *E*1ATP, and *E*1ADP boxes also contribute to sodium occlusion, whereas none of the species within the *E*2P box can occlude sodium.

Simulations of the model in Figure 11*A* using parameter values in Table 1 adequately reproduce the experimental findings (a complete list of parameters, including equilibrium constants for Na^+^ binding, is shown in Table S2). In particular, time courses show a rapid decrease in sodium occlusion as the enzyme remains in a steady state (Fig. 11*B*), during which *E*2P states—which do not occlude sodium—are predominant (*cf*., Fig. 10*A*). Besides, the duration of such a state increases with the concentration of ATP initially added and remains until all ATP is consumed. When considering Na^+^ occlusion as a function of [Na^+^] (Fig. 11*C*), simulated results correctly predict Na^+^ occlusion in equilibrium (with Mg^2+^, *cf*., *open circles* in Fig. 9*B*) as well as under steady-state conditions (*cf*., *gray circles* in Fig. 9*B*). The model also explains the increase in the affinity for Na^+^ and the considerable reduction in the maximum value of Na^+^ occluded in steady state compared with that in equilibrium (*cf*., Fig. 10*B*). Moreover, the steady-state equations derived predict that the relationship (steady state/equilibrium) between occlusion values as [Na^+^] tends to infinity approximately represents the fraction of the phosphorylated enzyme that exists as *E*1P in steady state, that is (*k*_21_ + *k*_dp3_)/(*k*_12_ + k_21_ + *k*_dp3_)—see Supporting information section.

**Table 1:** Summary of parameter values used for simulations shown in Figures 10*A* and 11, *B* and *C* of the model presented in Figure 11*A*

Parameter	Value	Reference
*k*_ATP_	15 μM^−1^ s^−1^	Heyse *et al.* (48) and Fedosova and Esmann (49) reported a value of 60 μM^−1^ s^−1^
*k*_−ATP_	2 s^−1^	Heyse *et al.* (48) reported a value of 1.64 s^−1^, within a range of 0.75–3 s^−1^. Fedosova and Esmann (49) reported 12 s^−1^
*k*_ADP_	15 μM^−1^ s^−1^	Assumed to be equal to *k*_ATP_
*k*_−ADP_	40 s^−1^	Estimated from a *K*_*d*_ of 3 μM reported by Fedosova and Esmann (50)
*k*_p_	200 s^−1^	Campos and Beaugé (51) and Heyse *et al.* (48)
*k*_dp_^a^	2.2 s^−1^	Mårdh and Lindahl (52) and Kaufman *et al.* (11, 14)
*k*_12_	328 s^−1^	Ganea *et al.* (35)
*k*_21_	66 s^−1^	Ganea *et al.* (35)

a *k*_dp_=*k*_dp0_=*k*_dp1_=*k*_dp2_=*k*_dp3_ (Table S2).

## Discussion

For decades, it has been fairly certain that Na^+^-occluded states take part in the Na^+^ transport pathway. Several research groups have already proposed that the Na^+^/K^+^-ATPase can occlude Na^+^ (21, 22, 23, 24); however, they based their conclusions on experiments involving enzyme states that do not exist in the ATPase transport cycle. These results never made it possible to discern whether Na^+^ occlusion occurs before (35, 36) or after (21, 22, 23) enzyme phosphorylation, and it is still accepted that Na^+^ is only occluded in *E*1P.

In this work, we show direct evidence of the existence of Na^+^-occluded intermediates of the Na^+^/K^+^-ATPase in noninhibited conditions. While the K^+^-occluded intermediates could be captured by rapid cooling and dilution of the enzyme (10, 11, 12, 13, 14, 17), the direct observation of the native Na^+^-occluded states required the utilization of a quench-flow approach using a specific reagent. EGCg allows rapid stabilization and isolation of the intermediates containing occluded Na^+^, both at equilibrium and during ATP hydrolysis. Its prompt binding prevents Na^+^ rapid deocclusion, without modifying the amount of occluded ^22^Na^+^ present in the reaction. The systematic failure to measure Na^+^ occlusion by the standard method of passage through an ion exchange resin column in unmodified preparations of the enzyme led to hypothesizing that the closure of the intracellular access and opening of the extracellular access might be concerted events (37). In such a case, the existence of states containing occluded Na^+^ would be too transient to be detected. The fact that it is possible to detect Na^+^-occluded intermediates highlights that Na^+^ transport is not a concerted mechanism.

Remarkably, EGCg increases Na^+^ binding affinity detected by eosin fluorescence, whereas it produces no difference in the affinity for occluded Na^+^; consequently, it can be inferred that EGCg does not significantly change the equilibrium between occluded and bound (but not occluded) Na^+^. The value of *K*_0.5_ for Na^+^ obtained from occlusion experiments contrasts with the submillimolar value obtained from eosin fluorescence experiments, a discrepancy that we have shown is only apparent if we consider cation occlusion to be a more complex process than a mere conformational change induced by cation binding.

The current knowledge, in accordance with the Albers–Post mechanism, suggests that Na^+^ occlusion should increase simultaneously with the formation of *E*1P, and no Na^+^ occlusion is expected in a nonphosphorylated enzyme. However, we found that Na^+^ becomes spontaneously occluded in the *E*1 dephosphorylated form of the Na^+^/K^+^-ATPase. This species has neither been reported in a fully functional enzyme before nor considered for explaining Na^+^ interaction with the enzyme.

The amount of occluded Na^+^ increases with Na^+^ concentration apparently along a rectangular hyperbola with a maximum stoichiometry of about three Na^+^ per enzyme unit, indicating that the equilibrium between the occluded and nonoccluded forms of the Na^+^-bound enzyme is shifted toward the former. It is worth noting that our results show lower affinity than those presented by Nyblom *et al.* (23) using an AlF_4_/ADP-inhibited preparation (*K*_0.5_ = 0.58 mM) and by Esmann and Skou (24) using an oligomycin-inhibited enzyme (*K*_0.5_ = 0.7 mM).

Although the hyperbolical shape of the curve might correspond to a system with three identical and independent sites for Na^+^, an inspection of the results at very low [Na^+^] reveals the existence of significant positive interactions between the sites. While in this work we have used models in which only *E*1Na_3_ can generate the occluded form *E*1(Na_3_), our results do not exclude the possibility of formation of minor fractions of occluded forms containing one or two Na^+^ ions, which would explain why we can measure occlusion at a concentration of the cation as low as 50 nM.

Our results are consistent with an ordered sequential binding mechanism, in agreement with previous crystallographic (38) and electrophysiological evidence (19, 20) of the strictly sequential nature of Na^+^ ions binding and release. Kanai *et al.* (38) proposed a sequential cooperative mechanism for the binding of Na^+^ in which there is only one pre-existing site, so-called *site III*; the other two sites would be formed after the previous one in the sequence is occupied by Na^+^. The specificity for Na^+^ would be explained if, because of its size, this cation but not K^+^ or its congeners can reach *site III* and produce the necessary changes to form the other sites.

The method we present also allowed measuring the evolution of species with occluded Na^+^ along the time course of ATP hydrolysis upon addition of ATP + Mg^2+^ to the enzyme suspension. We found that Na^+^-occluded species show a transient decrease—corresponding to the steady state of the Na^+^-ATPase reaction—which lasted the longer the higher the concentration of ATP. Such a decrease most probably reflects the formation of *E*2P and the disappearance of pre-existent Na^+^-occluded *E*1 forms since, during the Na^+^-ATPase reaction cycle, the limiting step is the dephosphorylation of *E*2P.

In this work, we isolated species of the Na^+^/K^+^-ATPase with tightly bound Na^+^ consistent with genuine occluded states as evidenced by (i) the observed stoichiometry, (ii) the fact that it is unaffected by extensive washing with high concentrations of unlabeled Na^+^ and K^+^—which would not be the case with simple equilibrium binding, (iii) the effects of Rb^+^, Mg^2+^, and unlabeled Na^+^, and (iv) the transient change in the distribution of species after the addition of ATP + Mg^2+^.

The knowledge of the existence of nonphosphorylated Na^+^-occluded forms of the Na^+^/K^+^-ATPase widens the capabilities of the Albers–Post model for explaining the Na^+^ transport pathway. We anticipate this will be the starting point for more sophisticated research about the involvement of Na^+^ occlusion in the transport pathway of the Na^+^/K^+^-ATPase and, in general, about cation transport through biological membranes. For instance, while in this study, we were able to measure the Na^+^ occluded in the enzyme that performs the Na^+^-ATPase reaction cycle, more studies should be carried out to determine the behavior of this property during the main mode of operation of the pump, that is, the exchange of Na^+^ for K^+^. Moreover, interest will arise for the obtention of structural data of nonphosphorylated states of the enzyme, and the search for molecules that affect the physiological cycle of the pump will refocus. Furthermore, being the Na^+^/K^+^-ATPase, a prototypical P-type ATPase, both our finding and methodology will encourage and make possible research on cation transport in other related enzymes.

## Experimental procedures

### Enzyme conditions

Na^+^/K^+^-ATPase (EC 7.2.2.13) was partially purified from pig kidneys as described elsewhere (39). The ouabain-sensitive specific activity at the time of preparation was 23 to 25 μmol of Pi per min per mg of protein, measured under optimal conditions (130 mM NaCl, 20 mM KCl, 3 mM ATP, and 4 mM MgCl_2_ in 25 mM imidazole/HCl, pH 7.4 at 37 °C). The maximum number of nucleotide sites in the preparations used in this work was estimated through phosphorylation experiments as 2.2 to 2.6 nmol per mg of protein. All incubations were performed at 25 °C in media containing 25 mM imidazole/HCl (pH 7.4 at 25 °C) and 0.25 mM EDTA. The concentrations of other components are indicated in each figure legend.

### Reagents

[^22^Na]NaCl (^22^Na^+^) was obtained from PerkinElmer. The fluorescent probe eosin-Y (eosin), as well as ATP, was from MilliporeSigma. ATP was obtained as imidazolium salt by passing the solution through a column containing the cation exchange AG MP50 resin (Bio-Rad) previously equilibrated with imidazole. EGCg was obtained from Aktin Chemicals. All other reagents were of analytical grade.

### Conformational changes

The conformational states of the Na^+^/K^+^-ATPase were studied using eosin, which exhibit high fluorescence signal for states in *E*1 and low fluorescence signal for states in *E*2 (40, 41). Protein (40 μg ml^−1^) was incubated with 0.4 μM eosin; the enzyme was kept in the dark at 25 °C throughout the experiments. Equilibrium measurements were performed with an FP-6500 spectrofluorometer (Jasco); the excitation and emission wavelengths were 520 and 540 nm, respectively, with a band-pass of 3 nm. Measurements of the time course of fluorescence were performed with an SX-18MV stopped-flow reaction analyzer (Applied Photophysics); the excitation wavelength was 520 nm, and emitted light was filtered through an OG550 filter (Schott); in each experiment, 1000 data points were collected and between five and seven experimental traces were averaged to evaluate each time course. As EGCg was found to quench the fluorescence of both bound and free eosin, signal values were corrected to compare between conditions with different [EGCg]. Fluorescence values were divided by the *F*_*∞*_ value obtained from fitting Equation S1 to the data. The underlying idea is that when [Na^+^] tends to infinity, all the enzymes will be in the *E*1 conformation and, therefore, maximal fluorescence values should coincide in all conditions.

### Measurement of occluded Na^+^

^22^Na^+^ occlusion was measured using a rapid filtration technique (42). Briefly, reactions were carried out in an SFM4 rapid-mixing apparatus (Bio-Logic); to stop the reactions and isolate the species with occluded Na^+^, the reaction medium was injected into a QWC with a cellulose-esters filter (Millipore) through which an ice-cold (ca. 4 °C) washing solution (25 mM imidazole/HCl, pH 7.4 at 4 °C, 120 mM NaCl, and 30 mM KCl) was flowing at a rate of about 40 ml s^−1^. The membrane in the QWC quantitatively retains the enzyme and allows the washing out of free and weakly bound Na^+^. Unless otherwise stated, the washing step was performed using 250 ml of washing solution containing 1 mM EGCg. In this setup, EGCg is typically present only in the washing solution. After washing, the filter was removed, dried, and counted for radioactivity (Fig. 3). Radioactivity counting was performed for enough time for errors to be less than 5%, regardless of the amount of cpm contained in the sample. Unless otherwise stated, protein concentration in enzyme suspensions was 100 μg ml^−1^. Equilibrium occlusion of Na^+^ was attained by incubating the enzyme for at least 20 min.

### Data analysis

The equations were fitted to the experimental data by a nonlinear regression procedure using OriginPro 2017 and MS Excel (MS Office 365). When necessary, to discriminate between several equations that fitted similarly well the experimental results, we used the F-test (43) or the second-order Akaike information criterion (44). Parameters are expressed as value ± standard error. To fit the kinetic model to experimental data, we used the freeware program COPASI 4.37 (http://copasi.org/) (45).

## Data availability

Most of the data described are contained within the article. Any additional data or further description will be shared upon request to the corresponding author.

## Supporting information

This article contains supporting information (46, 47).

## Conflict of interest

The authors declare that they have no conflicts of interest with the contents of this article.

## References

[1] Skou J.C., Esmann M. (1992). The Na,K-ATPase. J. Bioenerg. Biomembr.

[2] Kaplan J.H. (2002). Biochemistry of Na,K-ATPase. Annu. Rev. Biochem..

[3] Morth J.P., Pedersen B.P., Buch-Pedersen M.J., Andersen J.P., Vilsen B., Palmgren M.G. (2011). A structural overview of the plasma membrane Na+,K+-ATPase and H+-ATPase ion pumps. Nat. Rev. Mol. Cell Biol..

[4] Siegel G.J., Albers R.W. (1967). Sodium-potassium-activated adenosine triphosphatase of Electrophorus electric organ. IV. Modification of responses to sodium and potassium by arsenite plus 2,3-dimercaptopropanol. J. Biol. Chem..

[5] Albers R.W. (1967). Biochemical aspects of active transport. Annu. Rev. Biochem..

[6] Post R.L., Hegyvary C., Kume S. (1972). Activation by adenosine triphosphate in the phosphorylation kinetics of sodium and potassium ion transport adenosine triphosphatase. J. Biol. Chem..

[7] Beaugé L.A., Glynn I.M. (1979). Occlusion of K ions in the unphosphorylated sodium pump. Nature.

[8] Glynn I.M. (1993). Annual review prize lecture. 'All hands to the sodium pump'. J. Physiol..

[9] Mattle D., Sitsel O., Autzen H.E., Meloni G., Gourdon P., Nissen P. (2013). On allosteric modulation of P-type Cu(+)-ATPases. J. Mol. Biol..

[10] Forbush B. (1987). Rapid release of 42K and 86Rb from an occluded state of the Na,K-pump in the presence of ATP or ADP. J. Biol. Chem..

[11] Kaufman S.B., González-Lebrero R.M., Schwarzbaum P.J., Nørby J.G., Garrahan P.J., Rossi R.C. (1999). Are the states that occlude rubidium obligatory intermediates of the Na(+)/K(+)-ATPase reaction?. J. Biol. Chem..

[12] González-Lebrero R.M., Kaufman S.B., Montes M.R., Nørby J.G., Garrahan P.J., Rossi R.C. (2002). The occlusion of Rb(+) in the Na(+)/K(+)-ATPase. I. The identity of occluded states formed by the physiological or the direct routes: Occlusion/deocclusion kinetics through the direct route. J. Biol. Chem..

[13] Gonzalez-Lebrero R.M., Kaufman S.B., Garrahan P.J., Rossi R.C. (2002). The Occlusion of Rb(+) in the Na(+)/K(+)-ATPase. II. The effects of Rb(+), Na(+), Mg2(+), or ATP on the equilibrium between free and occluded Rb(+). J. Biol. Chem..

[14] Kaufman S.B., González-Lebrero R.M., Rossi R.C., Garrahan P.J. (2006). Binding of a single Rb+ increases Na+/K+-ATPase, activating dephosphorylation without stoichiometric occlusion. J. Biol. Chem..

[15] Morth J.P., Pedersen B.P., Toustrup-Jensen M.S., Sorensen T.L., Petersen J., Andersen J.P. (2007). Crystal structure of the sodium-potassium pump. Nature.

[16] Castillo J.P., Rui H., Basilio D., Das A., Roux B., Latorre R. (2015). Mechanism of potassium ion uptake by the Na(+)/K(+)-ATPase. Nat. Commun..

[17] Faraj S.E., Centeno M., Rossi R.C., Montes M.R. (2019). A kinetic comparison between E2P and the E2P-like state induced by a beryllium fluoride complex in the Na,K-ATPase. Interactions with Rb. Biochim. Biophys. Acta Biomembr.

[18] Glynn I.M., Hoffman J.F. (1971). Nucleotide requirements for sodium-sodium exchange catalysed by the sodium pump in human red cells. J. Physiol..

[19] Holmgren M., Wagg J., Bezanilla F., Rakowski R.F., De Weer P., Gadsby D.C. (2000). Three distinct and sequential steps in the release of sodium ions by the Na+/K+-ATPase. Nature.

[20] Gadsby D.C., Bezanilla F., Rakowski R.F., De Weer P., Holmgren M. (2012). The dynamic relationships between the three events that release individual Na⁺ ions from the Na⁺/K⁺-ATPase. Nat. Commun..

[21] Glynn I.M., Hara Y., Richards D.E. (1984). The occlusion of sodium ions within the mammalian sodium-potassium pump: its role in sodium transport. J. Physiol..

[22] Vilsen B., Andersen J.P., Petersen J., Jorgensen P.L. (1987). Occlusion of 22Na+ and 86Rb+ in membrane-bound and soluble protomeric alpha beta-units of Na,K-ATPase. J. Biol. Chem..

[23] Nyblom M., Poulsen H., Gourdon P., Reinhard L., Andersson M., Lindahl E. (2013). Crystal structure of Na+, K(+)-ATPase in the Na(+)-bound state. Science.

[24] Esmann M., Skou J.C. (1985). Occlusion of Na+ by the Na,K-ATPase in the presence of oligomycin. Biochem. Biophys. Res. Commun..

[25] Fersht A. (2017).

[26] Ochiai H., Takeda K., Soeda S., Tahara Y., Takenaka H., Abe K. (2009). Epigallocatechin-3-gallate is an inhibitor of Na+, K(+)-ATPase by favoring the E1 conformation. Biochem. Pharmacol..

[27] Esmann M. (1991). Oligomycin interaction with Na,K-ATPase: oligomycin binding and dissociation are slow processes. Biochim. Biophys. Acta.

[28] Esmann M. (1992). Properties of oligomycin-induced occlusion of Na+ by detergent-solubilized Na,K-ATPase from pig kidney or shark rectal gland. Biochim. Biophys. Acta.

[29] Skou J.C., Esmann M. (1983). The effects of Na+ and K+ on the conformational transitions of (Na+ + K+)-ATPase. Biochim. Biophys. Acta.

[30] Esmann M. (1994). Influence of Na+ on conformational states in membrane-bound renal Na,K-ATPase. Biochemistry.

[31] Sachs J.R. (1988). Interaction of magnesium with the sodium pump of the human red cell. J. Physiol..

[32] Pilotelle-Bunner A., Cornelius F., Sebban P., Kuchel P.W., Clarke R.J. (2009). Mechanism of Mg2+ binding in the Na+,K+-ATPase. Biophys. J..

[33] Apell H.J., Hitzler T., Schreiber G. (2017). Modulation of the Na,K-ATPase by magnesium ions. Biochemistry.

[34] Laursen M., Yatime L., Nissen P., Fedosova N.U. (2013). Crystal structure of the high-affinity Na+K+-ATPase-ouabain complex with Mg2+ bound in the cation binding site. Proc. Natl. Acad. Sci. U. S. A..

[35] Ganea C., Babes A., Lüpfert C., Grell E., Fendler K., Clarke R.J. (1999). Hofmeister effects of anions on the kinetics of partial reactions of the Na+,K+-ATPase. Biophys. J..

[36] Or E., David P., Shainskaya A., Tal D.M., Karlish S.J. (1993). Effects of competitive sodium-like antagonists on Na,K-ATPase suggest that cation occlusion from the cytoplasmic surface occurs in two steps. J. Biol. Chem..

[37] Forbush B. (1988). Overview: occluded ions and Na, K-ATPase. Prog. Clin. Biol. Res..

[38] Kanai R., Ogawa H., Vilsen B., Cornelius F., Toyoshima C. (2013). Crystal structure of a Na+-bound Na+,K+-ATPase preceding the E1P state. Nature.

[39] Klodos I., Esmann M., Post R.L. (2002). Large-scale preparation of sodium-potassium ATPase from kidney outer medulla. Kidney Int..

[40] Skou J.C., Esmann M. (1981). Eosin, a fluorescent probe of ATP binding to the (Na+ + K+)-ATPase. Biochim. Biophys. Acta.

[41] Montes M.R., Gonzalez-Lebrero R.M., Garrahan P.J., Rossi R.C. (2006). Eosin fluorescence changes during Rb+ occlusion in the Na+/K(+)-ATPase. Biochemistry.

[42] Rossi R.C., Kaufman S.B., Gonzalez Lebrero R.M., Norby J.G., Garrahan P.J. (1999). An attachment for nondestructive, fast quenching of samples in rapid-mixing experiments. Anal. Biochem..

[43] Motulsky H., Christopoulos A. (2003). *Fitting Models to Biological Data Using Linear and Nonlinear Regression. A Practical Guide to Curve Fitting*.

[44] Burnham K.P., Anderson D.R. (2002).

[45] Hoops S., Sahle S., Gauges R., Lee C., Pahle J., Simus N. (2006). COPASI--a COmplex PAthway SImulator. Bioinformatics.

[46] Fiske C.H., Subbarow Y. (1925). The colorimetric determination of phosphorus. J Biol Chem.

[47] Cha S. (1968). A simple method for derivation of rate equations for enzyme-catalyzed reactions under the rapid equilibrium assumption or combined assumptions of equilibrium and steady state. J Biol Chem.

[48] Heyse S., Wuddel I., Apell H.J., Stürmer W. (1994). Partial reactions of the Na,K-ATPase: determination of rate constants. J. Gen. Physiol..

[49] Fedosova N.U., Esmann M. (2007). Nucleotide binding to Na,K-ATPase: pK values of the groups affecting the high affinity site. Biochemistry.

[50] Fedosova N.U., Esmann M. (2004). Nucleotide-binding kinetics of Na,K-ATPase: cation dependence. Biochemistry.

[51] Campos M., Beaugé L. (1992). Effects of magnesium and ATP on pre-steady-state phosphorylation kinetics of the Na+,K(+)-ATPase. Biochim. Biophys. Acta.

[52] Mårdh S., Lindahl S. (1977). On the mechanism of sodium- and potassium-activated adenosine triphosphatase. Time course of intermediary steps examined by computer simulation of transient kinetics. J. Biol. Chem..

