# Supplementary material for: Measurements of Na+-occluded intermediates during the catalytic cycle of the Na+/K+-ATPase provide novel insights into the mechanism of Na+ transport
Source: J Biol Chem. 2022 Dec 17;299(2):102811. doi: 10.1016/j.jbc.2022.102811 (PMC9860123; doi:10.1016/j.jbc.2022.102811)
Supplement: Supplemental information [file mmc1.pdf]

SUPPORTING INFORMATION

Measurements of Na^+-occluded intermediates during the catalytic cycle of the Na^+/K^+-ATPase provide novel insights into the mechanism of Na^+ transport

Santiago E Faraj, Wanda M Valsecchi, Mariela Ferreira-Gomes, Mercedes Centeno, Elina Malén Saint Martin, Natalya U Fedosova, Juan Pablo FC Rossi, Mónica R Montes & Rolando C Rossi

EGCg directly interacts with the Na^+/K^+-ATPase, modifying ATPase activity, enzyme conformation and cation binding capabilities.

Figure S1 shows that Na^+/K^+-ATPase activity decreases with [EGCg]. Fitting of a Hill equation to the data results in a $K_{0.5}$ of 1.10 ± 0.03 μM and an n value of 2.1 ± 0.1.

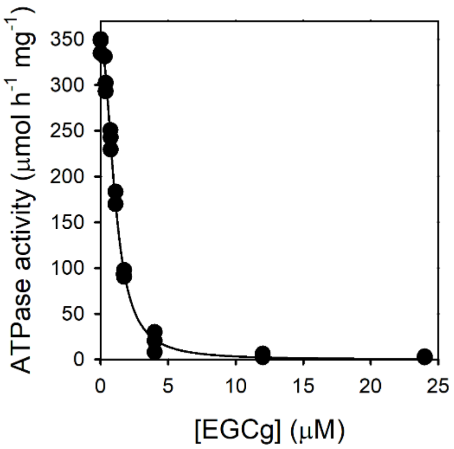

Figure S1. Inhibition of Na^+/K^+-ATPase activity by EGCg. ATPase activity was measured at varying [EGCg] at 25 ºC by following the release of Pi from ATP using the procedure by Fiske and Subbarow (1). Experiments were performed with 10 µg of protein per ml, and the composition of the incubation medium was 150 mM NaCl, 20 mM KCl, 3.0 mM $MgCl_2$ and 0.25 mM EDTA in 25 mM imidazole/HCl (pH 7.4 at 25 °C). Reactions were started by the addition of 2 mM ATP. Non-enzymatic ATP hydrolysis was subtracted from each measurement value. Three independent experiments are superimposed. The continuous line is the best global fitting of a Hill equation to the data.

We also assessed the effect of EGCg on the conformational state. We found that EGCg can bind to the enzyme in the absence of any ligand and stabilize the $E1$ conformation (Figure S2). Note that no traces of Na^+ (which well might shift the equilibrium to $E1$ with the concomitant increase of eosin fluorescence) are found in the EGCg.

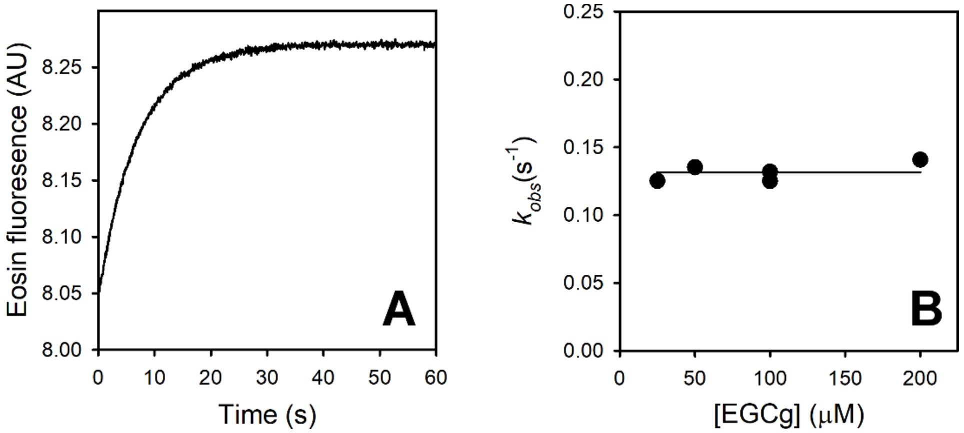

Figure S2. Kinetics of EGCg binding to the Na^+/K^+-ATPase. Eosin fluorescence change upon adding EGCg to an enzyme suspension incubated with 50 μM RbCl. (*A*) Time course acquired in the presence of 100 μM EGCg. (*B*) Observed rate coefficient (k_{obs}) values as a function of [EGCg], obtained by fitting an exponential function of time to traces like that shown in panel *A*. All reaction media also contained 25 mM imidazole/HCl (pH 7.4 at 25 °C) and 0.25 mM EDTA.

To assess the effect of EGCg on the enzyme affinity for Na^+ we measured eosin fluorescence under equilibrium conditions, at varying concentrations of EGCg and Rb^+ (Figure S3).

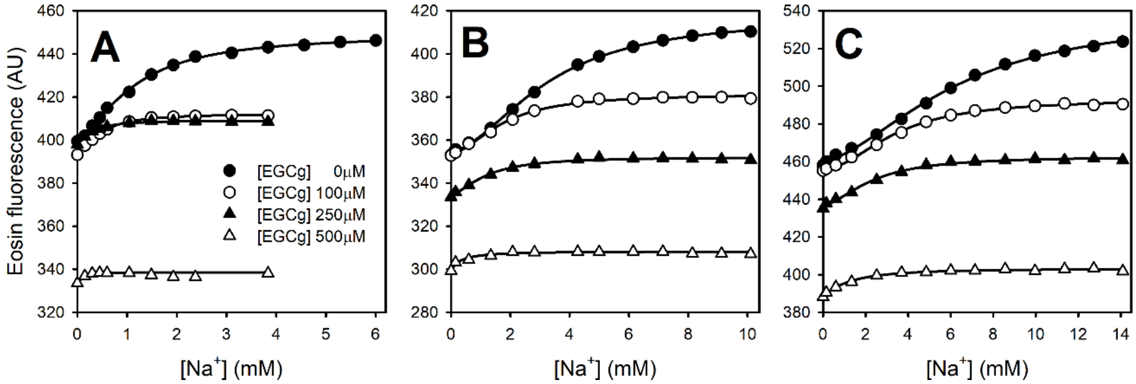

Figure S3. Effect of EGCg on Na^+ affinity of Na^+/K^+-ATPase. Eosin fluorescence in equilibrium experiments performed in an enzyme suspension as a function of [NaCl], in the presence of (*A*) 25, (*B*) 100 and (*C*) 250 μM RbCl. All reaction media also contained 25 mM imidazole/HCl (pH 7.4 at 25 °C) and 0.25 mM EDTA.

Note that when EGCg is present, both bound and free eosin fluorescence (F) signals tend to drop (Figure S3). Therefore, a comparison of Na^+ titration curves required normalizing them to their maximum changes (F/F_∞, cf. Figure 2 in the main manuscript). The fluorescent signal can be described by:

$$\mathrm{F} = \frac{F_0\,K1\,K2 + F_1\,K2\left[\mathrm{Na}^+\right] + F_\infty\left[\mathrm{Na}^+\right]^2}{K1\,K2 + K2\left[\mathrm{Na}^+\right] + \left[\mathrm{Na}^+\right]^2} \qquad \text{Eq. S1}$$

where F_0, F_1 and F_∞ are fluorescence signal parameters, and $K1$ and $K2$ are apparent dissociation constants for Na^+. Besides, the $K_{0.5}$ is defined as the [Na^+] that produces half of the signal change:

$$\mathrm{F_0} + \frac{\mathrm{F_\infty} - \mathrm{F_0}}{2} = \frac{F_0\, K1\, K2 + F_1\, K2\, K_{0.5} + F_\infty\, K_{0.5}^2}{K1\, K2 + K2\, K_{0.5} + K_{0.5}^2} \qquad \text{Eq. S2}$$

Solving for F_1 in Eq. S2 and replacing in Eq. S1, we obtain

$$\mathrm{F} = \frac{2\, F_0\, K_{0.5}\, K1\, K2 + F_0 \left(K_{0.5}^2 + K_{0.5}\, K2 - K1\, K2\right) [\mathrm{Na^+}] + F_\infty\, [\mathrm{Na^+}] \left[K1\, K2 + K_{0.5}\, (K2 + 2\, [\mathrm{Na^+}]) - K_{0.5}^2\right]}{2\, K_{0.5}\, [K1\, K2 + [\mathrm{Na^+}]\, (K2 + [\mathrm{Na^+}])]} \qquad \text{Eq. S3}$$

which allows estimating the value of F_∞ and $K_{0.5}$ by fitting to the data in Figure S3. The values obtained for the parameters are shown in Table S1.

Table S1. Summary of best-fitting parameters of Eq. S3 to data in Figure S3.

**[RbCl] (μM)**		**[EGCg] (μM)**			
		**0**	**100**	**200**	**500**
**25**	F_0 (AU)	399 ± 1	393.3 ± 0.3	398.0 ± 0.4	333.5 ± 0.3
	F_∞ (AU)	449 ± 3	412 ± 1	410 ± 1	338.4 ± 0.4
	$K_{0.5}$ (mM)	1.07 ± 0.09	0.43 ± 0.04	0.28 ± 0.04	0.10 ± 0.04
	$K1$ (mM)	2.0 ± 1.0	0.8 ± 0.3	0.3 ± 0.4	$12200 \pm 15{\cdot}10^6$
	$K2$ (mM)	0.9 ± 0.3	0.6 ± 0.4	0.3 ± 0.6	0 ± 0.001
**100**	F_0 (AU)	354.2 ± 0.5	353 ± 1	335 ± 1	300 ± 1
	F_∞ (AU)	417 ± 4	382 ± 3	352 ± 2	308 ± 3
	$K_{0.5}$ (mM)	3.1 ± 0.3	1.7 ± 0.2	1.1 ± 0.2	0.3 ± 0.2
	$K1$ (mM)	19 ± 21	7 ± 8	3 ± 2	0.3 ± 0.2
	$K2$ (mM)	0.7 ± 0.6	0.6 ± 0.5	1 ± 1	5 ± 76
**250**	F_0 (AU)	460 ± 1	456 ± 1	436 ± 1	389 ± 1
	F_∞ (AU)	537 ± 16	494 ± 4	463 ± 3	403 ± 3
	$K_{0.5}$ (mM)	6 ± 1	3.4 ± 0.3	2.3 ± 0.3	1.1 ± 0.4
	$K1$ (mM)	25 ± 50	18 ± 28	6 ± 7	1.4 ± 0.8
	$K2$ (mM)	2 ± 3	0.8 ± 0.9	1 ± 1	4 ± 15

Composition of washing solution

We investigated the effect of adding EGCg, Na^+, and K^+ in the washing solution on $^{22}Na^+$ retention on filters. A marginal influence of the content of Na^+ and K^+ in the solution was observed (Figure S4). Values of $^{22}Na^+$ retained on the filters were slightly lower when the washing solution contained both Na^+ and K^+ than when one of the cations was missing. In turn, the latter condition led to slightly lower values than using washing solutions with neither Na^+ nor K^+. Based on these results, the washing solution usually contained 1 mM EGCg, 120 mM NaCl and 30 mM KCl.

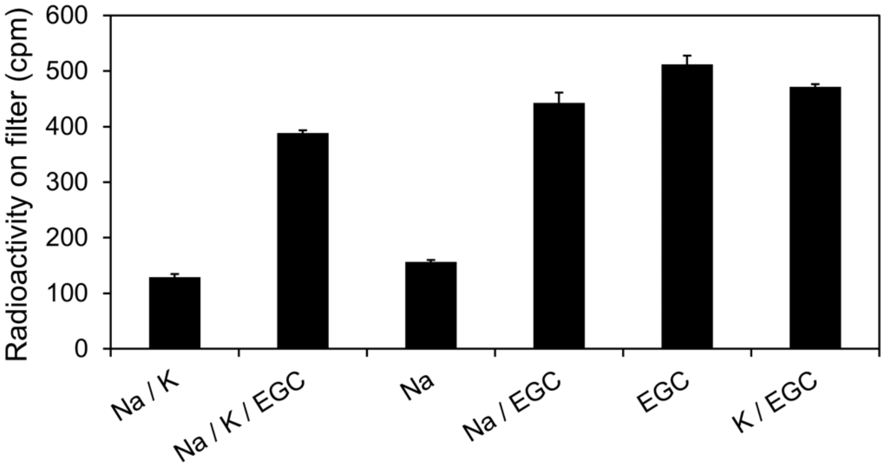

Figure S4. Effect of EGCg on $^{22}Na^+$ retention. Na^+/K^+-ATPase in equilibrium with 12 mM $^{22}Na^+$ was injected into the QWC and washed with buffer supplemented with or without 120 mM NaCl and/or 30 mM KCl and/or 1 mM EGCg. All reaction media also contained 25 mM imidazole/HCl (pH 7.4 at 25 °C) and 0.25 mM EDTA. Error bars represent ± 1 S.D.

Occlusion blanks

A question that arises is whether data obtained when the washing solution was not supplemented with EGCg genuinely evaluate Na^+ occlusion, or if it is an artifact, namely, the slope *m* of non-specific $^{22}Na^+$ binding. We analyzed the amount of radioactivity non-specifically retained on filters when the injected enzyme was either active or thermally inactivated (Figure S5).

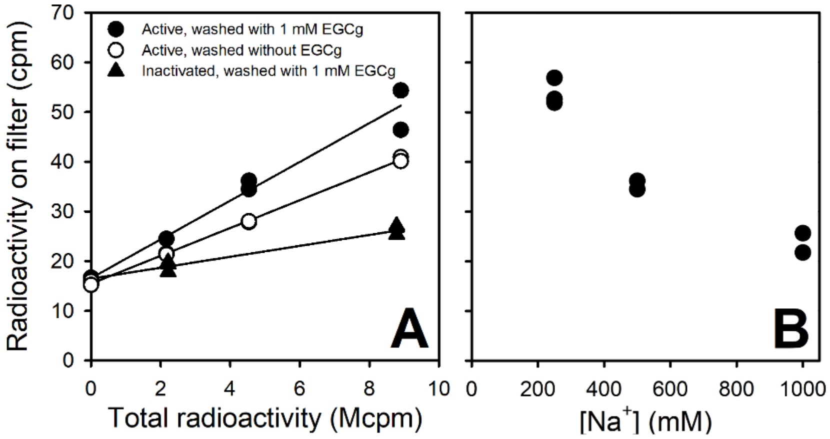

Figure S5. Unspecific binding of Na^+. (*A*) Na^+/K^+-ATPase in equilibrium with 500 mM $^{22}Na^+$ at varying specific activities (expressed as the total radioactivity that passed through the filter, Mcpm), was injected into the QWC and washed with or without 1 mM EGCg. (*B*) Na^+/K^+-ATPase in equilibrium with varying [NaCl] and 4.5 Mcpm of $^{22}Na^+$, was injected into the QWC and washed with 1 mM EGCg. All reaction media also contained 25 mM imidazole/HCl (pH 7.4 at 25 °C) and 0.25 mM EDTA.

Assays conducted with inactivated enzyme showed a *per se* effect of EGCg. Blanks performed using a thermally inactivated enzyme, showed to be insufficient to completely account for unspecific retention of $^{22}Na^+$, as evidenced by an unrelieved linear component (cf. Figure 5A). We suspect that such an additional component is related to the fact that blanks performed with inactivated enzyme might underestimate unspecific binding given that heating the enzyme most probably alters several components present in the preparation. However, once that contribution is subtracted, the amount of tightly bound Na^+ does not differ significantly between experiments in which EGCg was absent or present in the incubation medium (Figure 5B). It is worth noting that data obtained when the washing solution was not supplemented with EGCg does not indicate genuine Na^+ occlusion, as evidenced by the almost identical results when the enzyme was incubated with a great excess of unlabeled NaCl (Figure S5A), which allows showing unspecific $^{22}Na^+$ binding by an unaltered preparation.

Retained $^{22}Na^+$ is proportional to the concentration of Na^+/K^+-ATPase

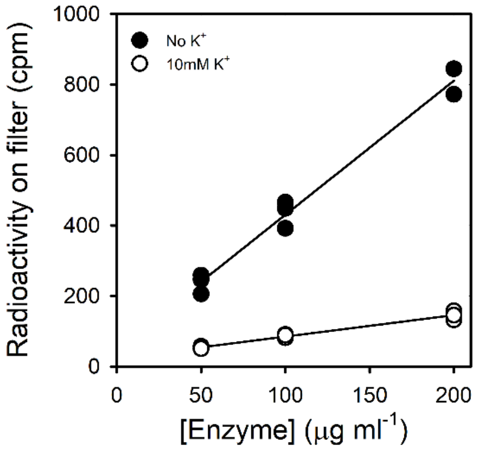

Figure S6. Effect of [enzyme] on $^{22}Na^+$ retention. Na^+/K^+-ATPase in equilibrium with 1 mM $^{22}Na^+$, with or without 10 mM K^+, was injected into the QWC and washed with buffer supplemented with 120 mM NaCl, 30 mM KCl and 1 mM EGCg. All reaction media also contained 25 mM imidazole/HCl (pH 7.4 at 25 °C) and 0.25 mM EDTA.

Effects of Rb^+ and Mg^{2+} on occluded Na^+

Total radioactivity on filters counted in experiments performed to assay the effect of Rb^+ and Mg^{2+} on Na^+ occlusion are shown in Figure S7.

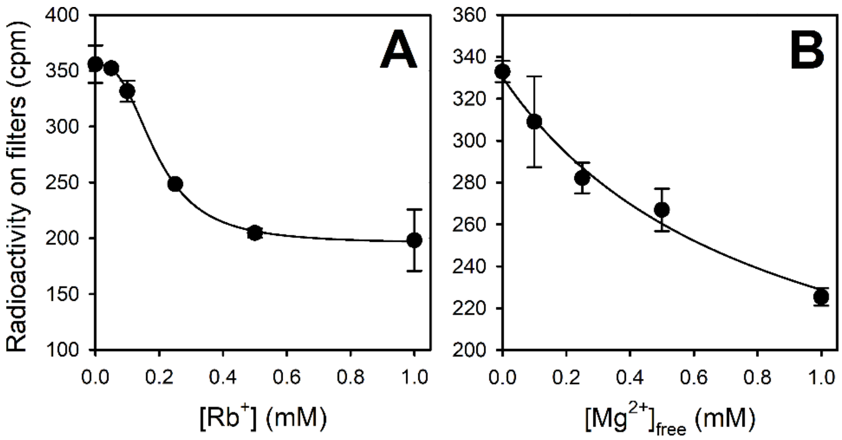

Figure S7. Effect of Rb^+ and Mg^{2+} on occluded Na^+. Equilibrium Na^+ occlusion (as total radioactivity retained on filters) at 6 mM NaCl as a function of (*A*) Rb^+ or (*B*) free Mg^{2+} concentrations. All reaction media also contained 25 mM imidazole/HCl (pH 7.4 at 25 °C) and 0.25 mM EDTA. Error bars represent ± 1 S.D.

Continuous lines are the best fitting of Eq. S4 and Eq.S5 to the data in Figure S7 A and B, respectively.

$$\text{Radioactivity} = \frac{A\left[\text{Rb}^{+}\right]^{\text{n}}}{{K_{0.5}}^{n} + \left[\text{Rb}^{+}\right]^{\text{n}}} + A_{\infty} \qquad \text{Eq. S4}$$

$$\text{Radioactivity} = \frac{B\left[\text{Mg}^{2+}\right]}{K_{0.5} + [\text{Mg}^{2+}]} + B_{\infty} \qquad \text{Eq. S5}$$

Percentage of the total change in cpm shown in Figure 9 A and B was calculated using Eq. S6 and Eq.S7, respectively.

$$\text{Occluded Na}^{+}\ (\%) = 100 \times \frac{\text{Radioactivity} - A_{\infty}}{A} \qquad \text{Eq. S6}$$

$$\text{Occluded Na}^{+}\ (\%) = 100 \times \frac{\text{Radioactivity} - B_{\infty}}{B} \qquad \text{Eq. S7}$$

Na^+-ATPase cycling model

Figure S8 is a more detailed model of the scheme presented in Figure 11A in the main text. Among the species in the $E1$ATP box, only $E1$ATP(Na_3) can undergo phosphorylation through k_p, and among those in the $E2$P box, only $E2PNa_3$ can revert to $E1$P(Na_3) through k_{21}, while all species within the $E2$P box can undergo dephosphorylation ($E2$P through k_{dp0}, $E2$PNa through k_{dp1}, $E2PNa_2$ through k_{dp2}, and $E2PNa_3$ through k_{dp3}).

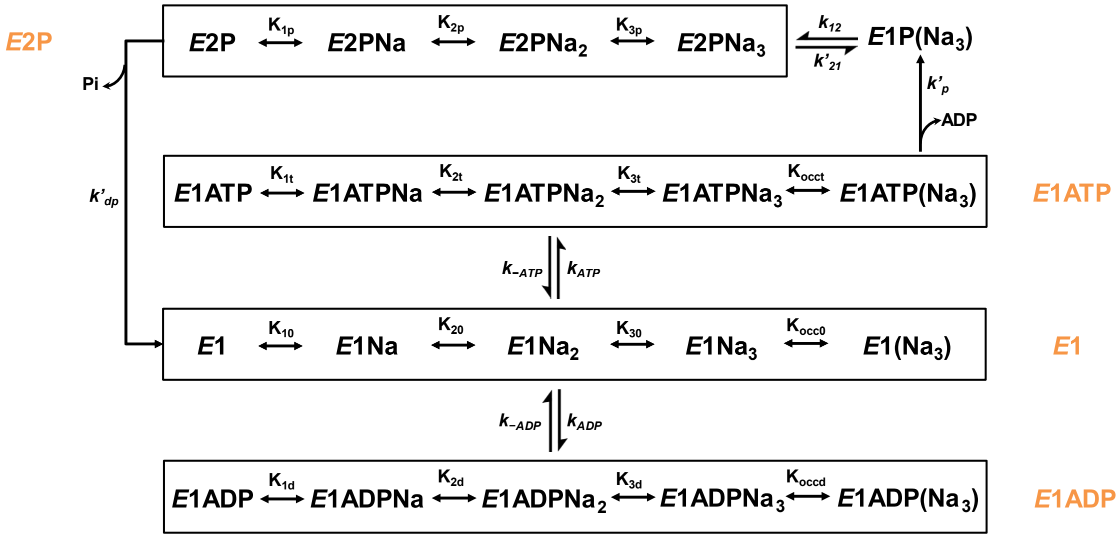

Figure S8. Model of the Na^+-ATPase cycle. A complete version of the model presented in Figure 11A in the main text, showing equilibriums between Na^+-holding species. Occluded Na^+ is shown between brackets.

Apparent rate constants k'_p, k'_{21}, and k'_{dp} are defined as the following linear combinations (2):

$$k'_p = k_p\, f_{E1\mathrm{ATP(Na_3)}} \qquad \text{Eq. S8}$$

$$k'_{21} = k_{21}\, f_{E2\mathrm{PNa_3}} \qquad \text{Eq. S9}$$

$$k'_{dp} = k_{dp0}\, f_{E2\mathrm{P}} + k_{dp1}\, f_{E2\mathrm{PNa}} + k_{dp2}\, f_{E2\mathrm{PNa_2}} + k_{dp3}\, f_{E2\mathrm{PNa_3}} \qquad \text{Eq. S10}$$

The total occluded Na^+ is calculated as:

$$\mathrm{Na_{occ}} = 3\left\{f_{E1\mathrm{(Na_3)}}\boxed{E1} + f_{E1\mathrm{ATP(Na_3)}}\boxed{E1\mathrm{ATP}} + f_{E1\mathrm{ADP(Na_3)}}\boxed{E1\mathrm{ADP}} + [E1\mathrm{P(Na_3)}]\right\} \qquad \text{Eq. S11}$$

where the concentration value of each box is defined by:

$$\boxed{E1} = [E1] + [E1\mathrm{Na}] + [E1\mathrm{Na_2}] + [E1\mathrm{Na_3}] + [E1\mathrm{(Na_3)}] \qquad \text{Eq. S12}$$

$$\boxed{E1\mathrm{ATP}} = [E1\mathrm{ATP}] + [E1\mathrm{ATPNa}] + [E1\mathrm{ATPNa_2}] + [E1\mathrm{ATPNa_3}] + [E1ATP(Na_3)] \qquad \text{Eq. S13}$$

$$\boxed{E1\mathrm{ADP}} = [E1\mathrm{ADP}] + [E1\mathrm{ADPNa}] + [E1\mathrm{ADPNa_2}] + [E1\mathrm{ADPNa_3}] + [E1ADP(Na_3)] \quad \text{Eq. S14}$$

The fractions of each species are defined by the following equations:

$$f_{E1(\mathrm{Na_3})} = \frac{K_{occ0}[\mathrm{Na^+}]^3}{K_{10}K_{20}K_{30} + K_{20}K_{30}[\mathrm{Na^+}] + K_{30}[\mathrm{Na^+}]^2 + [\mathrm{Na^+}]^3 + K_{occ0}[\mathrm{Na^+}]^3} \quad \text{Eq. S15}$$

$$f_{E1\mathrm{ATP(Na_3)}} = \frac{K_{occt}[\mathrm{Na^+}]^3}{K_{1t}K_{2t}K_{3t} + K_{2t}K_{3t}[\mathrm{Na^+}] + K_{3t}[\mathrm{Na^+}]^2 + [\mathrm{Na^+}]^3 + K_{occt}[\mathrm{Na^+}]^3} \quad \text{Eq. S16}$$

$$f_{E1\mathrm{ADP(Na_3)}} = \frac{K_{occd}[\mathrm{Na^+}]^3}{K_{1d}K_{2d}K_{3d} + K_{2d}K_{3d}[\mathrm{Na^+}] + K_{3d}[\mathrm{Na^+}]^2 + [\mathrm{Na^+}]^3 + K_{occd}[\mathrm{Na^+}]^3} \quad \text{Eq. S17}$$

$$f_{E2\mathrm{P}} = \frac{K_{1p}K_{2p}K_{3p}}{K_{1p}K_{2p}K_{3p} + K_{2p}K_{3p}[\mathrm{Na^+}] + K_{3p}[\mathrm{Na^+}]^2 + [\mathrm{Na^+}]^3} \quad \text{Eq. S18}$$

$$f_{E2\mathrm{PNa}} = \frac{K_{2p}K_{3p}[\mathrm{Na^+}]}{K_{1p}K_{2p}K_{3p} + K_{2p}K_{3p}[\mathrm{Na^+}] + K_{3p}[\mathrm{Na^+}]^2 + [\mathrm{Na^+}]^3} \quad \text{Eq. S19}$$

$$f_{E2\mathrm{PNa_2}} = \frac{K_{3p}[\mathrm{Na^+}]^2}{K_{1p}K_{2p}K_{3p} + K_{2p}K_{3p}[\mathrm{Na^+}] + K_{3p}[\mathrm{Na^+}]^2 + [\mathrm{Na^+}]^3} \quad \text{Eq. S20}$$

$$f_{E2\mathrm{PNa_3}} = \frac{[\mathrm{Na^+}]^3}{K_{1p}K_{2p}K_{3p} + K_{2p}K_{3p}[\mathrm{Na^+}] + K_{3p}[\mathrm{Na^+}]^2 + [\mathrm{Na^+}]^3} \quad \text{Eq. S21}$$

Tables 1 and S2 summarize the values assigned to the parameters to produce the simulations of the model shown in Figure 11A in the main manuscript.

Table S2. Summary of parameter values used for simulations shown in Figures 9A and 10B-C of the model presented in Figure 11A.

Parameter	Value
k_{ATP}	15 μM^{-1} s^{-1}
k_{-ATP}	2 s^{-1}
k_{ADP}	15 μM^{-1} s^{-1}
k_{-ADP}	40 s^{-1}
k_p	200 s^{-1}
k_{dp0}	2.2 s^{-1}
k_{dp1}	2.2 s^{-1}
k_{dp2}	2.2 s^{-1}
k_{dp3}	2.2 s^{-1}
k_{12}	328 s^{-1}
k_{21}	66 s^{-1}

Parameter	Value
$K_{10}= K_{1t}= K_{1d}$	0.5 mM
$K_{20}= K_{2t}= K_{2d}$	2 mM
$K_{30}= K_{3t}= K_{3d}$	1000 mM
$K_{occ0}= K_{occt}= K_{occd}$	100
$K_{1p}= K_{2p} = K_{3p}$	5 mM

The model was also used to produce the simulations of the time courses shown in Figure S9 below.

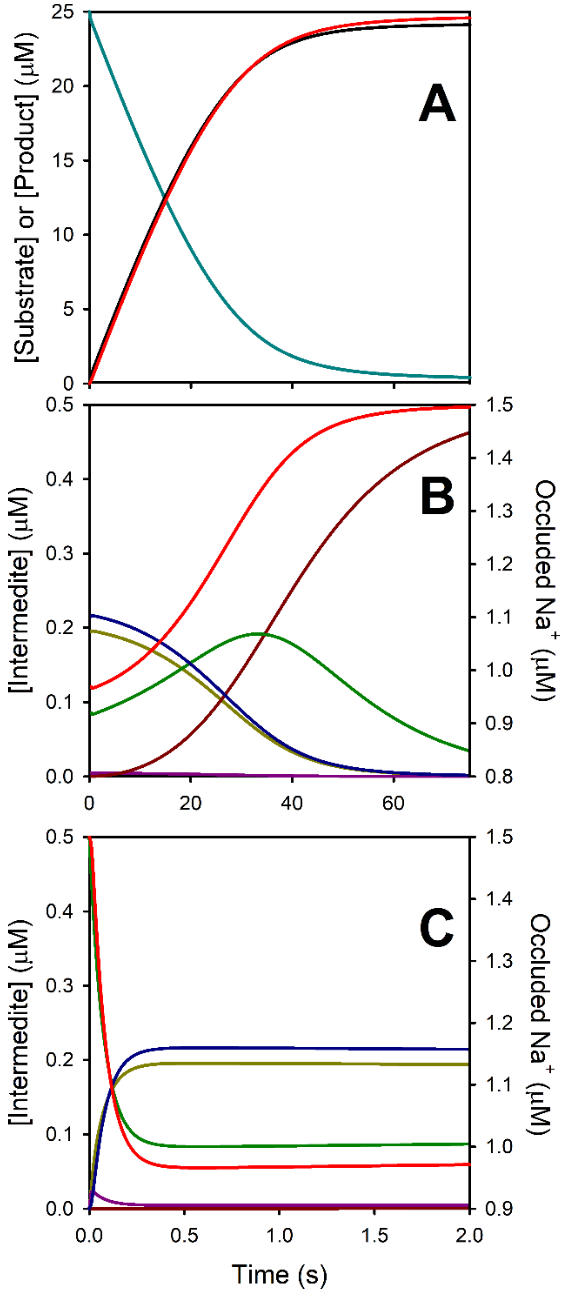

Figure S9. Time course of ATP hydrolysis. Concentration of different species during ATP hydrolysis simulated using the model shown in Figure 11 for the parameters in Table 1 and [ATP] = 25 μM and Na^+ = 6 mM. (*A*) Concentrations of ATP (▬), ADP (▬) and Pi (▬). The linear component at the beginning of the reaction represents the *initial rate*. (*B-C*) Concentration of the intermediates in the model (Figure 11), *E*1PNa (▬), *E*1ATP (▬), *E*1ADP (▬), *E*1 (▬) and *E*2P (▬), and occluded Na^+ (▬). The almost constant component at the beginning of the reaction (0.5-2.5 s) represents the initial *steady state*. Note that the initial rate of ATP consumption and ADP and Pi production (A) takes place while the reaction elapses under the initial steady-state conditions (B and C). As these conditions dissipate, the reaction progresses towards equilibrium.

Inclusion of the new findings in the Albers-Post model quantitatively predicts the steady-state drop in occluded Na^+

According to the model in Figure 11A, the steady-state velocity $\left(v_{SS}\right)$ can be defined as

$$v_{SS} = \boxed{E2\mathrm{P}}\,k'_{\mathrm{dp}} = \left[E1\mathrm{P}\left(\mathrm{Na}_3\right)\right]k_{12} - \boxed{E2\mathrm{P}}\,k'_{21} \qquad \text{Eq. S22}$$

From the expression in Eq. S22,

$$\left[E1\mathrm{P}\left(\mathrm{Na}_3\right)\right] = \frac{\boxed{E2\mathrm{P}}\left(k'_{21} + k'_{\mathrm{dp}}\right)}{k_{12}} \qquad \text{Eq. S23}$$

The fraction of the concentration of phosphoenzyme $\left(\left[E1\mathrm{P}\left(\mathrm{Na}_3\right)\right] + \boxed{E2\mathrm{P}}\right)$ that exists as $E1\mathrm{P}\left(\mathrm{Na}_3\right)$ will be

$$\frac{\left[E1\mathrm{P}\left(\mathrm{Na}_3\right)\right]}{\left[E1\mathrm{P}\left(\mathrm{Na}_3\right)\right] + \boxed{E2\mathrm{P}}} = \frac{k'_{21} + k'_{\mathrm{dp}}}{k_{12} + k'_{21} + k'_{\mathrm{dp}}} \qquad \text{Eq. S24}$$

As [Na^+] tends to infinity, the equilibrium value of occluded Na^+ is proportional to the total amount of enzyme. If k'_{dp} is small, most of the enzyme will remain in phosphorylated forms $\left(\left[E1\mathrm{P}\left(\mathrm{Na}_3\right)\right] + \boxed{E2\mathrm{P}}\right)$, hence values of occluded Na^+ will be proportional to $\left[E1\mathrm{P}\left(\mathrm{Na}_3\right)\right]$. Therefore, as Na^+ tends to infinity, the relationship between the values of occluded Na^+ in steady state and equilibrium (1.0 nmol mg^{-1} / 6.0 nmol mg^{-1} = 0.167) will approximately represent the fraction of the phosphorylated enzyme that exists as $E1P(Na_3)$ in steady state (Eq. S24). Eq. S24 using values in Table 1 predicts a value of 0.172, which is consistent with the experimental relationship. Note that, as [Na^+] tends to infinity, $k'_{dp} \rightarrow k_{dp3}$ and $k'_{21} \rightarrow k_{21}$.

References

1. Fiske, C. H., and Subbarow, Y. (1925) The colorimetric determination of phosphorus. *J Biol Chem* **66**, 375-400

2. Cha, S. (1968) A simple method for derivation of rate equations for enzyme-catalyzed reactions under the rapid equilibrium assumption or combined assumptions of equilibrium and steady state. *J Biol Chem* **243**, 820-825
